# Safety, tolerability, pharmacokinetics, and pharmacodynamics of zapnometinib: results from a phase I clinical study

**DOI:** 10.3389/fphar.2026.1743230

**Published:** 2026-04-17

**Authors:** Stephan Stenglein, Julian Volk, Yvonne Füll, Christian Reh, Steffen Haffner, Sebastian Canisius, Tim Overend, Oliver Planz

**Affiliations:** 1 Institute of Immunology, University and University Hospital of Tübingen, Tübingen, Germany; 2 Atriva Therapeutics GmbH, Martinsried, Germany; 3 Nuvisan GmbH, Neu-Ulm, Germany; 4 ACliRA Consulting ApS, Tønder, Denmark

**Keywords:** mek inhibitor, pharmacokinetics, phase I, safety, tolerability, zapnometinib

## Abstract

**Introduction:**

Zapnometinib (ATR-002) is a selective MEK inhibitor designed to modulate the MAPK/ERK pathway, which plays a key role in viral infections and inflammatory diseases. Clinical characterization of its safety, tolerability, pharmacokinetics (PK), and pharmacodynamics (PD) is essential to support further development. This Phase I clinical trial was registered under EudraCT number 2021-005225-25.

**Methods:**

This was a Phase I, single-center, randomized, double-blind, placebo-controlled trial conducted in healthy adults. The study included three parts: a single ascending dose (SAD) phase, a multiple ascending dose (MAD) phase, and a drug–drug interaction (DDI) as well as a food-drug interaction (FDI) phase. In the SAD phase, 42 participants received single oral doses of 600, 900, 1,200, or 1,500 mg. In the MAD phase, 29 participants received daily oral doses of 900, 1,200, or 1,500 mg for 7 days. The DDI phase assessed the effect of zapnometinib on CYP2C8 and CYP2C9 activity using repaglinide and celecoxib as probe substrates.

**Results:**

Zapnometinib was well tolerated with no serious adverse events reported. Most treatment-emergent adverse events (TEAEs) were mild to moderate, including gastrointestinal symptoms (e.g., diarrhea, nausea) and headache. PK analysis showed dose-proportional increases in C_max_ and AUC, with a notable food effect that increases bioavailability. PD evaluation demonstrated significant MEK inhibition, evidenced by reduced ERK phosphorylation.

**Discussion/Conclusion:**

This phase 1 study demonstrates that zapnometinib has a favorable safety and tolerability profile, predictable pharmacokinetics, and potent pharmacodynamic activity. The results support further clinical development of zapnometinib for therapeutic indications involving dysregulated MAPK/ERK signaling.

## 1 Introduction

The mitogen-activated protein kinase/extracellular signal–regulated kinase (MAPK/ERK) signaling cascade is a central regulator of cell proliferation, differentiation, and survival. Dysregulation of this pathway has been implicated in a wide range of pathological conditions, including cancer, autoimmune and inflammatory disorders, and viral infections (Lavoie et al., 2020; Dhillon et al., 2007; Zhang and Liu, 2002; Arthur and Ley, 2013; Planz, 2013). Within this cascade, MEK1 and MEK2 are dual-specificity kinases that activate ERK1/2 through phosphorylation (Roskoski, 2012; Pearson et al., 2001). Because of this key regulatory role, MEK has emerged as an inhibitable therapeutic target for attenuating aberrant MAPK/ERK activity.

Zapnometinib (ATR-002) is a selective MEK inhibitor that has demonstrated strong antiviral and immunomodulatory potential in preclinical and clinical studies (Laure et al., 2020; Schreiber et al., 2022b; Koch-Heier et al., 2024; Rohde et al., 2023; Füll et al., 2025). In contrast to traditional direct-acting antivirals, zapnometinib targets a host signaling pathway exploited by many RNA viruses. Several RNA viruses, including influenza virus, exploit the Raf/MEK/ERK signaling cascade to support replication and enhance pathogenesis (Liu and Luo, 2024; Ludwig et al., 2023; Ludwig et al., 2003). Notably, influenza A virus shows particularly strong dependence on this pathway, underscoring its vulnerability to MEK inhibition (Hoffmann et al., 2023). By modulating MAPK/ERK activity, zapnometinib suppresses viral replication and mitigates hyperinflammatory responses, including the cytokine storm-like syndromes observed in severe influenza and COVID-19 (Pinto et al., 2011; Schreiber et al., 2022b; Tang et al., 2020; Ludwig et al., 2023). These dual antiviral and host-directed effects underscore its potential as a versatile therapeutic candidate.

Despite the extensive preclinical evidence, clinical pharmacology data for zapnometinib remain limited. First-in-human studies are essential to establish its safety and tolerability, to characterize pharmacokinetics (PK) and pharmacodynamics (PD). Zapnometinib has already advanced to Phase II clinical evaluation (Rohde et al., 2023), following an initial Phase I study that established its basic safety, tolerability, and pharmacokinetic profile. However, several clinically relevant aspects remained insufficiently addressed in the earlier trial, particularly the effects of food on drug absorption and potential drug–drug interactions (DDIs). To address these open questions, we conducted an additional Phase I study in healthy adults with a focus on safety, tolerability, PK, PD, and the influence of food–drug interactions (FDIs) and DDIs on zapnometinib exposure.

In contrast to direct-acting antivirals, where the pharmacodynamic effect can be directly quantified by reduction of viral replication, the situation for host-directed agents is less straightforward as they exert effects via modulation of host pathways rather than direct viral inhibition (von Delft et al., 2023). For compounds such as zapnometinib, which act by inhibiting host kinases exploited by viruses, the pharmacodynamic readout is not the immediate antiviral effect itself but rather the degree and duration of target inhibition. Consequently, a more refined understanding of drug–target interaction kinetics becomes essential. Parameters such as the extent of target engagement investigating MEK inhibition provide critical mechanistic insight into how sustained inhibition can be achieved *in vivo*. Evaluating this parameter for zapnometinib is expected to offer mechanistic insights and contribute to positioning it within the broader class of MEK inhibitors.

The study used single ascending dose (SAD) and multiple ascending dose (MAD) designs to characterize dose–exposure, dose–pharmacodynamic (MEK inhibition), and dose–safety relationships across escalating dose levels, and included drug–drug interaction (DDI) assessments with repaglinide and celecoxib as CYP2C8 and CYP2C9 probe substrates, respectively. Since MEK inhibitors are known to interfere with cellular proliferation, concerns have been raised that such effects might also influence immune cell homeostasis. Previous preclinical and clinical studies with zapnometinib have not provided evidence of clinically relevant immunosuppression at the investigated doses. *In vitro* studies with zapnometinib confirmed good tolerability without cytotoxicity in combination treatments (Schreiber et al., 2022a). Preclinical pharmacokinetic/pharmacodynamic investigations in animals and human volunteers showed consistent target engagement without adverse impacts on immune parameters in the intended doses (Koch-Heier et al., 2022). Moreover, in the Phase II RESPIRE trial (NCT0477604 and EudraCT 2020-004206-59) in hospitalized patients with COVID-19, the incidence of adverse events and key immunological markers like C-reactive protein (CRP) did not significantly differ between zapnometinib and placebo arms (Rohde et al., 2023). Nevertheless, we considered it important to explicitly address this aspect in the present trial, since higher doses of zapnometinib were used here. Therefore, we evaluated whether leukocyte and lymphocyte counts were reduced under treatment, and we monitored CRP levels in zapnometinib-treated subjects compared with placebo. Thus, in the present study, these evaluations not only informed dose selection and safety monitoring strategies for future clinical trials but also provided additional reassurance that zapnometinib does not adversely affect immune cell homeostasis or inflammatory markers, thereby supporting its further development and potential use in combination therapies.

This study assessed the safety and tolerability of zapnometinib and explored aspects of target engagement, contributing to a foundational understanding of its clinical pharmacological properties and supporting its further development for therapeutic indications with unmet medical needs associated with MAPK/ERK pathway dysregulation, including severe viral infections such as COVID-19.

## 2 Methods

### 2.1 Objectives, participants, and oversight

The zapnometinib Phase 1 trial (EudraCT Number: 2021-005225-252525) was a three-part study designed to evaluate the safety, tolerability, PK, and PD of zapnometinib in healthy volunteers, conducted in a specialized Phase 1 clinical unit with enhanced monitoring capabilities. The study consisted of SAD, MAD, FDI and DDI components. It was conducted in compliance with Good Clinical Practice (GCP) guidelines and approved by the responsible independent ethics committee (Ethik-Kommission der Bayerischen Landesärztekammer, München, Germany). All participants gave written informed consent before undergoing any study procedures.

The primary objective was to determine the safety profile and identify a well-tolerated dosing range, thereby providing essential information for the design of subsequent Phase 2 trials in diseases driven by dysregulated MAPK/ERK signaling, including viral infections and inflammatory diseases. Healthy male and female volunteers aged 18–55 years, with a body mass index (BMI) of 18.0–29.9 kg/m^2^, were eligible to participate. Exclusion criteria included a history of chronic illness, use of concomitant medications affecting drug metabolism, or prior participation in investigational drug studies within 30 days. Additional eligibility requirements are detailed in the Supplementary Material.

Key operational procedures included blinding methodology for placebo-controlled cohorts, stringent monitoring for adverse events, and strict adherence to study protocols for consistent data collection. Oversight responsibilities and methods, including regulatory compliance and data monitoring processes, are summarized in Supplementary Data S1.

### 2.2 Drug product

Zapnometinib (ATR-002) drug product was formulated as immediate-release oral tablets containing 150 mg of the active pharmaceutical ingredient (API). The formulation was optimized for stability and reproducible absorption characteristics under both fasted and fed condition. For the SAD and MAD phases, the tablets were administered orally with water under fasting conditions, except in the FDI cohort of the SAD phase, where the tablets were taken with a standardized high-fat, high-calorie meal (U.S. Food and Drug Administration, 2022). In the DDI phase, zapnometinib was co-administered with probe drugs (repaglinide for CYP2C8 and celecoxib for CYP2C9) to evaluate potential drug-drug interactions. The placebo tablets matched the zapnometinib drug product in appearance, weight, and inert excipient composition, maintaining blinding integrity throughout the study.

All zapnometinib and placebo drug products were manufactured under Good Manufacturing Practice (GMP) conditions to ensure quality and consistency. They were stored under controlled temperature and humidity conditions as specified in the trial protocol. Detailed information regarding the composition, quality control (QC), and manufacturing processes of the drug substance and drug product is provided in Supplementary Data S2.

### 2.3 Intervention and dosing

SAD Phase: Participants were randomized into four dose cohorts: 600 mg, 900 mg, 1,200 mg, and 1,500 mg of zapnometinib or placebo. Each participant received a single oral dose under fasting conditions; additionally, the first cohort also included a crossover design to evaluate food-drug interactions (fasted vs. fed states). A minimum washout period of 10 days was maintained between treatments for the crossover group.

MAD Phase: Participants were randomized into three dose cohorts: 900 mg, 1,200 mg, and 1,500 mg. They received a single daily dose of zapnometinib or placebo for 7 consecutive days.

DDI Phase: The potential effect of zapnometinib on CYP2C8 and CYP2C9 enzyme activity was evaluated using probe substrates repaglinide (0.5 mg) and celecoxib (100 mg). Celecoxib was selected based on its established role in clinical DDI evaluations, favorable safety and tolerability in healthy subjects, and practical advantages over alternatives such as S-warfarin, including a shorter half-life and lower bleeding risk. Subjects received zapnometinib (1,500 mg) with and without the probe drugs.

### 2.4 SAD, MAD, DDI, and FDI study schema

The SAD portion of the trial was a four-cohort, double-blind, placebo-controlled, randomized study to evaluate zapnometinib at single doses of 600 mg, 900 mg, 1,200 mg, and 1,500 mg. Each cohort included 10 participants, with 8 receiving the study drug and two receiving placebo under fasting conditions between 8 a.m. and 10 a.m. in the morning. Safety metrics, including vital signs (VS), electrocardiograms (ECGs), and adverse events (AEs), were recorded at baseline, on the dosing day, and during follow-up visits. Serial blood and urine samples were collected for PK analysis at predefined intervals. PD assessments included assessing the inhibition of ERK phosphorylation in peripheral blood mononuclear cells (PBMCs) to confirm MEK pathway engagement. A full safety and PK review was conducted prior to dose escalation for each subsequent cohort. The effect of food on zapnometinib absorption was also assessed in the first cohort using a crossover design. Participants received the same dose of zapnometinib under fasted and fed conditions (following a standardized high-fat, high-calorie meal), separated by a washout period of 10 days.

The MAD portion was a three-cohort, double-blind, placebo-controlled, randomized study assessing zapnometinib at daily doses of 900 mg, 1,200 mg, and 1,500 mg administered for 7 consecutive days. Each cohort included 10 participants, with 8 receiving the study drug and two receiving placebo, again always at approximately the same time in the morning (between 8 a.m. and 10 a.m.). Safety metrics and AE monitoring were conducted daily during the treatment period and during follow-up. PK blood samples were collected at multiple timepoints on Days 1 and 7 and at selected intervals on intervening days to assess accumulation and steady-state kinetics. PD assessments, including ERK phosphorylation inhibition, were performed to evaluate sustained MEK pathway inhibition during repeated dosing.

The design of the DDI and FDI assessments was guided by relevant regulatory recommendations for the clinical evaluation of pharmacokinetic interactions and food effects. In particular, the study design, selection of probe substrates, and timing of pharmacokinetic sampling were aligned with guidance on the investigation of drug interactions issued by the European Medicines Agency (European Medicines Agency, 2012). The DDI study evaluated the effects of zapnometinib (1,500 mg single dose) on CYP2C8 and CYP2C9 enzyme activities using repaglinide (0.5 mg) and celecoxib (100 mg) as probe substrates, respectively. For repaglinide: 11 participants received 2 single doses of 1,500 mg zapnometinib and 2 single doses of 0.5 mg repaglinide each. Zapnometinib was administered for two consecutive days to evaluate reversible CYP2C8 inhibition; as no mechanism-based inhibition or induction potential was identified in preclinical assessments, steady-state dosing was not required in accordance with regulatory guidance. For celecoxib: 11 participants received 4 single doses of 1,500 mg zapnometinib and 2 single doses of 100 mg celecoxib each. Blood samples were collected at predefined intervals to assess changes in the PK profiles of the probe drugs (e.g., C_max_, AUC_0-inf_, AUC_0-tlast_) and to calculate geometric mean ratios and confidence intervals (CIs) for drug interactions.

The FDI portion of the trial evaluated the effect of food on the PK of zapnometinib in the SAD phase, as described above. The 600 mg dose was chosen for the food-effect assessment as it was the starting SAD dose and provided a safe and clinically relevant exposure level, in line with regulatory recommendations to evaluate food effects early in development. In addition to PK endpoints, safety and AE monitoring were conducted to ensure tolerability under fed conditions.

For all study components, detailed safety reviews were conducted prior to dose escalations or additional administrations. Comprehensive data on PK, PD, and safety informed the selection of safe and well-tolerated doses for subsequent cohorts and future clinical phases.

### 2.5 Safety and tolerability

Key safety evaluations included monitoring physical examinations, clinical laboratory outcomes (e.g., hematology, biochemistry, and urinalysis), VS, and ECGs. Participants were closely monitored for any AEs and serious adverse events (SAEs) from the time of study drug administration until the end of the study. AEs were documented based on participant self-reporting and direct observations by study personnel of this specialized Phase 1 clinical unit. All participants were instructed to report any AEs to the study physician or research personnel at any time during the trial. The severity (mild, moderate, severe), duration, and potential relationship of AEs to the study drug were systematically recorded and assessed. Laboratory abnormalities were monitored for clinical significance and resolution during follow-up. SAE reporting followed regulatory requirements, including adherence to the U.S. Code of Federal Regulations (21 CFR Part 312.32) and Good Clinical Practice (GCP) guidelines. The data safety monitoring team conducted regular reviews to ensure participant safety, particularly prior to dose escalations in the SAD and MAD phases.

### 2.6 Bioanalytical methods

A validated bioanalytical method was used for all PK measurements of participant plasma containing K2EDTA as an anticoagulant. Samples were processed using protein precipitation followed by liquid chromatography-tandem mass spectrometry (LC-MS/MS) detection. Method development, calibration, and quality control (QC) of samples were performed using zapnometinib with an appropriate isotopically labeled internal standard to ensure accuracy and reproducibility. Sample stability was confirmed at storage temperatures of −20 °C and −80 °C, with the duration of stability deemed acceptable for up to 12 months at −20 °C and 24 months at −80 °C. These assessments ensured the integrity of study samples during storage and analysis.

The validated concentration range for zapnometinib in plasma spanned [e.g., 0.5 ng/mL (lower limit of quantitation, LLOQ) to 500 ng/mL (upper limit of quantitation, ULOQ)]. The method demonstrated precision with relative standard deviation (RSD%) values ≤ 15.0% and mean concentration within ±15.0% bias of the nominal concentration across QC sample levels.

Additional bioanalytical details, including chromatograms, calibration curves, and method validation parameters, are provided in Supplementary Data S3.

### 2.7 Pharmacokinetics

Blood samples for PK analysis were collected at predefined timepoints during the SAD, MAD, and DDI phases, according to the schedule of assessments and procedures. PK parameters were determined from plasma concentrations of zapnometinib using non-compartmental analysis. For the SAD phase, serial PK blood samples were collected for each participant receiving zapnometinib or placebo at thirteen timepoints: pre-dose, 0.5, 1, 2, 4, 6, 8, 12, 24, 36, 48, 72 and 96 h post-dose. Standard PK parameters calculated for the SAD cohorts included maximum plasma concentration (C_max_), time to maximum concentration (T_max_), area under the concentration-time curve ( ${\text{AUC}}_{0-\infty }$ and AUC_0–tlast_), clearance (CL), volume of distribution (Vz), and terminal elimination half-life (t_1/2_). Terminal elimination half-life (t_½_) was summarized using geometric mean values, consistent with the approximately log-normal distribution of PK parameters and aligned with the reporting of C_max_ and AUC.

For the MAD phase, serial PK blood samples were collected on Days 1 and 7 (first and last doses). For Day 1, at 11 timepoints: pre-dose, 1, 2, 3, 4, 5, 6, 9, 12, 15 and 24 h post-dose. For Day 7, at 7 timepoints: pre-dose, 12, 24, 36, 48, 72 and 96 h post-last dose. Additional blood samples were collected pre-dose and 1-h post-dose on Days 2–6 to monitor intermediate PK parameters. Standard PK parameters for the MAD cohorts included AUC_0–24h_, C_max_, AUC_0–tlast_, T_max_, Vz, CL, and accumulation ratios for C_max_ and AUC_0–tau_ (calculated using the ratio of parameters on Day 7 to Day 1).

For the DDI phase, PK sampling was performed at timepoints specific to the probe drugs. For repaglinide on day −2 and day 1: pre-dose, 0.25, 0.5, 0.75, 1, 1.25, 1.5, 2, 3, 4, 5, 6, 7, 8, 12 h post-dose and for celecoxib on day −4 and day 1: pre-dose, 0.5, 1, 1.5, 2, 2.5 3, 3.5 4, 5, 6, 9, 12, 15 and 24 h post-dose; on day −3 and day 2: 24 and 36 h post-dose; on day −2 and day 3: 48 h post-dose and on day −1 and day 4 72 h post dose (Supplementary Figure S1). Zapnometinib PK was analyzed concurrently to assess any reciprocal effects. PK parameters included ${\text{AUC}}_{0-\infty }$ , C_max_, and T_max_ for both the probe drugs and zapnometinib under single and combined administration conditions. For the FDI evaluation (conducted within the SAD phase), PK samples were collected following zapnometinib administration in fed and fasted states. Comparative analyses of plasma concentrations were performed to evaluate the impact of food on drug absorption and bioavailability, with standard parameters (C_max_, T_max_, ${\text{AUC}}_{0-\infty }$ , and AUC_0–tlast_) calculated for both conditions. All PK analyses used actual post-dose times relative to the start of dose administration. Additional details on the methodology for summarizing and reporting the PK data, as well as the statistical evaluations, are provided in Supplementary Data S4.

### 2.8 Pharmacodynamics

PD assessments were conducted during the SAD and MAD phases to evaluate the biological activity of zapnometinib and to confirm target engagement through MEK inhibition. For both the SAD and MAD phases, pharmacodynamic effects were measured using PBMCs collected at predefined timepoints. Blood samples of the participants were processed to isolate PBMCs, and levels of phosphorylated ERK (pERK) were quantified as a biomarker of MEK pathway activity. Samples were collected pre-dose, and at 1, 2, 4, 8, and 24 h post-dose during the SAD phase. For the MAD phase, samples were taken on Day 1 and Day 7 at similar time intervals as for the SAD phase, to assess sustained MEK inhibition after repeated dosing. The extent of pERK reduction was quantified relative to baseline levels, and expressed as the percent inhibition of ERK phosphorylation. These data were analyzed to establish the relationship between plasma concentrations of zapnometinib and its pharmacodynamic effects, providing insight into concentration-response and time-response relationships. Additional details on PBMC isolation, pERK quantification methods, and data analysis approaches, are provided in Supplementary Data S5.

### 2.9 Outcome measures

*Primary Endpoints*: Safety and tolerability, assessed via AE monitoring, clinical laboratory tests (hematology, biochemistry, and urinalysis), electrocardiograms, and vital signs.

*Secondary Endpoints*: PK: Plasma concentrations of zapnometinib and probe drugs were measured using validated LC-MS/MS methods. PK parameters included C_max_, t_max_, AUC, t_1/2_, and accumulation ratios (R_acc_); PD: MEK inhibition was evaluated by measuring the reduction of ERK phosphorylation in activated PBMCs.

### 2.10 Data analysis

This Phase I study was primarily descriptive and exploratory in nature; therefore, no confirmatory hypothesis-testing framework or formal power-based statistical testing was applied. Pharmacokinetic and pharmacodynamic data were summarized using descriptive statistics, including arithmetic or geometric mean values, measures of variability, and concentration–time profiles.

Non-compartmental pharmacokinetic analyses were performed using Phoenix WinNonlin® (Version 7.0 or higher). Descriptive and inferential data summaries were generated using SAS® (Version 9.4 or higher). Where appropriate, pharmacokinetic parameters were summarized using geometric means, reflecting the approximately log-normal distribution of pharmacokinetic data.

For selected exploratory assessments, model-based or comparative approaches were applied. Dose proportionality in the SAD part was evaluated using a power model on log-transformed exposure parameters. For food–drug interaction (FDI) and drug–drug interaction (DDI) evaluations, comparisons of log-transformed pharmacokinetic parameters were performed using analysis of variance (ANOVA) models to estimate geometric least-squares mean ratios and corresponding confidence intervals.

All data were listed and summarized descriptively by study part (SAD, MAD, and DDI). Scheduled measurements were used for statistical summaries unless considered unreliable due to technical reasons. Baseline values were defined as the last valid observation prior to first administration of investigational medicinal product. Missing data were not imputed.

Protocol deviations and withdrawals were documented and reviewed in data review meetings between sponsor and contract research organization.

## 3 Results

### 3.1 Study population

A total of 43 subjects, comprising 33 subjects on active treatment and 10 subjects on placebo, were enrolled to participate in the SAD part as follows: 10 subjects in the first and second SAD cohort (8 subjects on active treatment and 2 subjects on placebo each) and 11 subjects in the third and fourth SAD cohort (8 or 9 (for 1500mg)) subjects on active treatment and 3 subjects on placebo each), to allow for sentinel dosing (active vs. placebo) for these cohorts as a safety precaution. A total of 29 subjects, comprising 23 subjects on active treatment and 6 subjects on placebo, were enrolled to participate in the MAD part as follows: 10 subjects in each MAD cohort (8 subjects on active treatment (7 for 1500mg) and 2 subjects on placebo each). The SAD part was completed prior to commencement of the MAD part. After finalization of the SAD and MAD parts, potential DDI was investigated in 2 further cohorts (one cohort for repaglinide, one for celecoxib), which were to comprise 12 subjects each. All subjects received the active treatment of zapnometinib. Doses investigated were: 1,500 mg zapnometinib, 0.5 mg repaglinide and 100 mg celecoxib. Baseline characteristics of participants, including age, sex, and body mass index (BMI), were comparable across the dose cohorts. These characteristics are detailed in Table 1 for the SAD cohort and in Supplementary Tables S1, S2 for the SAD-DDI, and MAD cohorts. No significant differences in demographic or clinical baseline measures were observed between the treatment and placebo groups, supporting the comparability of the study populations.

**TABLE 1:** Baseline characteristics of participants in phase 1 SAD study.

Parameter		Placebo (N = 10)	600 mg^a^ (N = 8)	900 mg (N = 8)	1,200 mg (N = 8)	1,500 mg (N = 9)	Overall (N = 43)
Age (years)							
Mean (SD)		34.6 (9.91)	41.6 (7.95)	46.3 (7.40)	38.9 (4.16)	40.6 (10.27)	40.1 (8.88)
Med. (Range)		32.5 (22–52)	43.5 (31–49)	48.0 (33–53)	39.5 (31–44)	44.0 (22–55)	40.0 (22–55)
Height (cm)							
Mean (SD)		179.1 (9.83)	176.8 (8.40)	172.1 (11.43)	176.0 (4.00)	178.7 (9.68)	176.7 (9.00)
Med. (Range)		178.0 (164–199)	177.0 (166–188)	173.0 (155–187)	176.5 (170–181)	180.0 (162–194)	177.0 (155–199)
Weight (kg)							
Mean (SD)		80.16 (10.530)	83.51 (10.563)	76.00 (9.708)	80.95 (9.172)	80.57 (4.352)	80.24 (9.015)
Med. (Range)		76.90 (70.2–105.0)	83.85 (67.5–104.0)	74.70 (62.7–91.0)	79.25 (71.8–96.3)	79.5 (75.3–86.8)	78.00 (62.7–105.0)
BMI (kg/m^2^)							
Mean (SD)		24.98 (2.385)	26.66 (2.129)	25.70 (2.659)	26.08 (2.466)	25.36 (2.066)	25.71 (2.309)
Med. (Range)		24.25 (22.6–29.4)	26.00 (23.6–29.4)	26.25 (20.6–28.7)	25.60 (22.9–29.7)	26.20 (22.2–28.8)	25.70 (20.6–29.7)
Sex							
Female		1 (10.0)	2 (25.0)	3 (37.5)	0 (0.0)	1 (11.1)	7 (16.3)
Male		9 (90.0)	6 (75.0)	5 (62.5)	8 (100.0)	8 (88.9)	36 (83.7)
Ethnicity							
Not hispanic or latino		9 (90.0)	8 (100.0)	8 (100.0)	8 (100.0)	9 (100.0)	42 (97.7)
Hispanic or latino		1 (10.0)	0 (0.0)	0 (0.0)	0 (0.0)	0 (0.0)	1 (2.3)
Race							
White		10 (100.0)	8 (100.0)	8 (100.0)	8 (100.0)	9 (100.0)	43 (100.0)

^a^ Values are identical for ‘600 mg, fasted’ and ‘600 mg, fed’.

BMI, body mass index; Med = median; SD, standard deviation.

### 3.2 Safety and tolerability

#### 3.2.1 SAD part

In the SAD study, no deaths or SAEs occurred at any dose level (Table 2; Supplementary Tables S3). A total of 31 treatment-emergent adverse events (TEAEs) were reported in 17 of 43 participants (39.5%), with incidence increasing at higher doses. Of the 15 TEAEs considered possibly or probably related to zapnometinib, gastrointestinal disorders were most frequent (8 events in 7 participants), including diarrhea (2), nausea (2), and abnormal feces (2), observed exclusively at doses ≥900 mg. Nervous system disorders accounted for 7 TEAEs in 5 participants, including headache (3) and dizziness (2), mainly at the 1,500 mg dose. Other TEAEs (e.g., somnolence, taste disturbance, fatigue, warmth) were reported only in isolated cases. One subject at 600 mg experienced transient increases in amylase and lipase without clinical sequelae. Vital signs, ECGs, and laboratory values did not show clinically meaningful abnormalities, and deviations were judged unrelated to zapnometinib. A similar pattern of TEAEs was observed in the FDI group (Supplementary Tables S4).

**TABLE 2:** Overall summary of TEAEs–SAD part, dose escalation.

​	Placebo (N = 10)	600 mg (N = 8)	900 mg (N = 8)	1,200 mg (N = 8)	1,500 mg (N = 9)	Overall (N = 43)
Subjects with any TEAE	2 (20.0%)	2 (25.0%)	3 (37.5%)	3 (37.5%)	7 (77.8%)	17 (39.5%)
Subjects with any drug-related TEAE	2 (20.0%)	1 (12.5%)	2 (25.0%)	3 (37.5%)	7 (77.8%)	15 (34.9%)
Subjects with any mild TEAE	2 (20.0%)	2 (25.0%)	3 (37.5%)	3 (37.5%)	7 (77.8%)	17 (39.5%)
Subjects with any moderate TEAE	0 (0.0%)	0 (0.0%)	0 (0.0%)	0 (0.0%)	0 (0.0%)	0 (0.0%)
Subjects with any serious TEAE	0 (0.0%)	0 (0.0%)	0 (0.0%)	0 (0.0%)	0 (0.0%)	0 (0.0%)
Subjects with any TEAE leading to death	0 (0.0%)	0 (0.0%)	0 (0.0%)	0 (0.0%)	0 (0.0%)	0 (0.0%)
Subjects discontinued due to TEAE	0 (0.0%)	0 (0.0%)	0 (0.0%)	0 (0.0%)	0 (0.0%)	0 (0.0%)
Any TEAE	4	3	4	4	13	28
Drug-related TEAE	3	1	3	3	13	23
Mild TEAE	4	3	4	4	13	28
Serious TEAE	0	0	0	0	0	0
TEAE leading to death	0	0	0	0	0	0

TEAE, treatment-emergent adverse event; Drug-related TEAE: relationship rated as ‘possible’, ‘probable’ or ‘related’.

#### 3.2.2 DDI part

In the DDI study, no deaths, SAEs, or discontinuations occurred. In the repaglinide arm (CYP2C8), six subjects reported 18 TEAEs, most of which occurred during combination treatment with zapnometinib and repaglinide (13 events), with fewer during zapnometinib monotherapy (5 events); no TEAEs were reported with repaglinide alone. Reported events were predominantly gastrointestinal, such as abdominal pain and diarrhea, all of which were mild, transient, and resolved spontaneously (Supplementary Tables S5). In the celecoxib arm (CYP2C9), eleven subjects reported 49 TEAEs, primarily during zapnometinib monotherapy (18 events) and combination treatment (28 events), with only three events occurring during celecoxib alone. Gastrointestinal and nervous system disorders, including headache, were the most common. All events were mild, most of short duration (<24 h), and resolved without sequelae. Across both arms, the majority of TEAEs were considered at least possibly related to the investigational product (Supplementary Tables S6).

#### 3.2.3 MAD part

In the MAD study, no deaths or SAEs were reported, and none of the 29 participants discontinued due to adverse events. A total of 54 TEAEs occurred, of which 49 were mild and 5 moderate. Moderate events were observed only in zapnometinib-treated subjects. All 49 TEAEs considered at least possibly related to the investigational medicinal product (IMP) occurred in active treatment groups. Five TEAEs—cystitis, headache, oral herpes, seborrheic dermatitis, and toothache—required concomitant medication or procedures but were manageable and resolved.

The most frequent TEAEs were gastrointestinal (30 events in 10 subjects, e.g., abnormal feces, abdominal discomfort, nausea), almost exclusively in zapnometinib-treated subjects. Renal/urinary disorders (7 events in 5 subjects, e.g., urinary tract pain) and nervous system disorders (6 events in 4 subjects, e.g., headache, dizziness) were also common. All ECGs and vital signs were normal or showed non-significant deviations. Most laboratory values remained within reference ranges; nine abnormalities were classified as clinically significant, including recurrent positive occult blood in stool (3 times in one placebo subject; once in a subject at 1,200 mg). TEAEs were generally transient (max. duration ∼14.5 days) and resolved without intervention, except one placebo subject with persistent fecal occult blood at study end. Overall, zapnometinib was well tolerated across all doses tested in the MAD part (Table 3; Supplementary Tables S7).

**TABLE 3:** Overall summary of the TEAEs – MAD dose escalation.

​	Placebo (N = 6)	900 mg (N = 8)	1,200 mg (N = 8)	1,500 mg (N = 7)	Overall (N = 29)
Subjects with any TEAE	2 (33.3%)	5 (62.5%)	7 (87.5%)	3 (42.9%)	17 (58.6%)
Subjects with any drug-related TEAE	0 (0.0%)	5 (62.5%)	7 (87.5%)	3 (42.9%)	15 (51.7%)
Subjects with any mild TEAE	2 (33.3%)	4 (50.0%)	7 (87.5%)	3 (42.9%)	16 (55.2%)
Subjects with any moderate TEAE	0 (0.0%)	2 (25.0%)	2 (25.0%)	1 (14.3%)	5 (17.2%)
Subjects with any serious TEAE	0 (0.0%)	0 (0.0%)	0 (0.0%)	0 (0.0%)	0 (0.0%)
Subjects with any TEAE leading to death	0 (0.0%)	0 (0.0%)	0 (0.0%)	0 (0.0%)	0 (0.0%)
Subjects discontinued due to TEAE	0 (0.0%)	0 (0.0%)	0 (0.0%)	0 (0.0%)	0 (0.0%)
Any TEAE	3	12	27	12	54
Drug-related TEAE	0	12	26	11	49
Mild TEAE	3	10	25	11	49
Moderate TEAE	0	2	2	1	5
Serious TEAE	0	0	0	0	0
TEAE leading to death	0	0	0	0	0

TEAE, treatment-emergent adverse event; Drug-related TEAE: relationship rated as ‘possible’, ‘probable’ or ‘related’.

#### 3.2.4 Leukocyte counts and CRP levels

Given previous concerns that MEK inhibition might affect immune cell homeostasis, leukocyte counts and CRP levels were monitored in the MAD study, with a focus on the highest dose (1,500 mg). Over the 11-day observation period (8 treatment days +3 follow-up days), leukocyte counts remained within normal physiological ranges with no sustained or clinically relevant reductions (Figure 9A). Transient declines occurred in both placebo and zapnometinib groups but showed no drug-related trend. CRP levels were stable in both groups, with no indication of zapnometinib-associated systemic immunosuppression (Figures 3A,B). These findings support the conclusion that zapnometinib, even at the highest dose, does not adversely affect leukocyte homeostasis or systemic inflammatory markers. Full data across all dose groups are provided in the Supplementary Material (Supplementary Tables S8, S9).

Across all study parts (SAD, MAD, and DDI), zapnometinib was well tolerated at doses up to 1,500 mg. Most TEAEs were mild, transient, and predominantly gastrointestinal or nervous system related, with no deaths, SAEs, or discontinuations observed. Exploratory safety analyses further indicate that zapnometinib does not adversely affect leukocyte homeostasis or systemic inflammatory markers, supporting a favorable overall safety profile.

### 3.3 Pharmacokinetic evaluation

PK analyses of zapnometinib were performed to characterize plasma concentration–time profiles, dose proportionality, and elimination characteristics after single and multiple oral administrations. In addition, FDI and DDI assessments were conducted to evaluate the influence of external factors on zapnometinib exposure.

#### 3.3.1 SAD pharmacokinetics

PK assessments following single-dose administration under fasted conditions were conducted to determine plasma concentration–time profiles, peak exposure, and systemic drug exposure across increasing dose levels. These data provide the basis for evaluating dose proportionality of zapnometinib after single administration.

**FIGURE 1:**
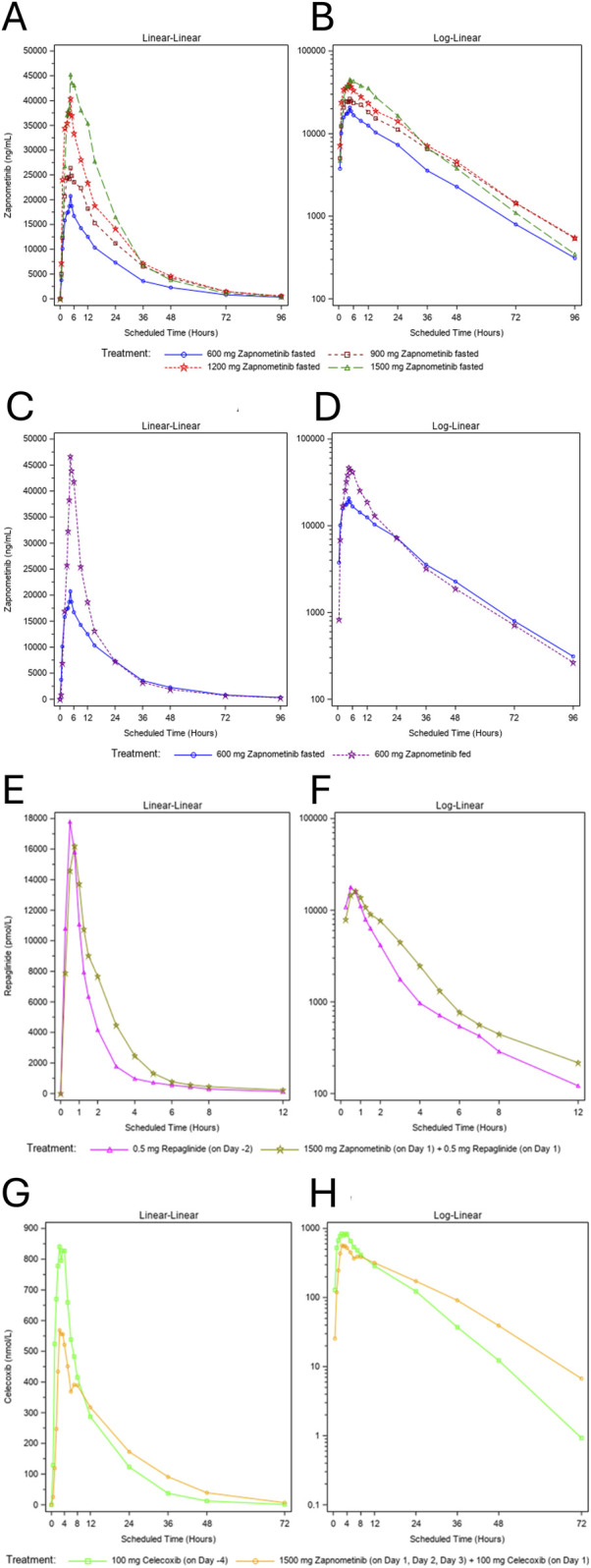
Mean Plasma Concentration-Time Profiles of zapnometinib - SAD Part. Dose Escalation. Plasma concentration–time profiles of zapnometinib following single ascending doses (SAD study). Data represent arithmetic mean plasma concentrations for each dose cohort. **(A,B)** Plasma concentration–time profiles on Day 1 after single oral doses of 600 mg, 900 mg, 1,200 mg, and 1,500 mg, shown on a linear scale **(A)** and a logarithmic scale **(B)**. Concentrations increased proportionately with dose and remained above the LLOQ (50 ng/mL) for all cohorts. **(C,D)** Arithmetic mean plasma concentration–time profiles of zapnometinib (600 mg) under fasted and fed conditions (food–drug interaction, FDI). Peak concentrations occurred later and were higher in the fed state (46.6 μg/mL at 4.5 h; ∼2.3-fold vs. fasted). Concentrations declined thereafter but remained above the LLOQ up to 96 h. Linear scale **(C)**; logarithmic scale **(D)**. **(E,F)** Arithmetic mean plasma concentration–time profiles of repaglinide (0.5 mg) administered alone or co-administered with 1,500 mg zapnometinib. Plasma concentrations increased rapidly post-dose, peaking at 0.500 h (17.3 nmol/L) when given alone and at 0.517 h (16.3 mol/L) when given in combination, before declining steadily but remaining above the LLOQ up to 12 h. Linear scale **(E)**; logarithmic scale **(F)**. **(G,H)** Arithmetic mean plasma concentration–time profiles of celecoxib (100 mg) administered alone or co-administered with 1,500 mg zapnometinib. Rapid absorption was observed with peak levels at 2.5 h post-dose (1.0 nmol/L) when given alone and at 3 h (0.6 nmol/L) when given in combination. Concentrations then declined, with quantifiable levels maintained over the 24-h interval. Linear scale **(G)**; logarithmic scale **(H)**.

Prior to administration, zapnometinib plasma concentrations were below the LLOQ. Following single-dose administration under fasted conditions, concentrations increased dose-dependently with peaks between 2.00 and 5.00 h across all cohorts. At ∼4.5 h post-dose, peak levels reached 20.7 μg/mL (600 mg), 26.4 μg/mL (900 mg), 40.4 μg/mL (1,200 mg), and 45.2 μg/mL (1,500 mg). Concentrations declined thereafter but remained above the LLOQ in all cohorts up to 96 h post-dose. The resulting concentration–time profiles from the SAD part are shown in Figure 1A (linear scale) and Figure 1B (log scale); pharmacokinetic parameters are summarized in Table 4. Median T_max_ ranged from 2.00 to 5.00 h. Geometric mean C_max_ increased from 21.0 μg/mL (600 mg) to 50.1 μg/mL (1,500 mg), and geometric mean ${\text{AUC}}_{0-\infty }$ from 439.8 to 974.9 h μg/mL, consistent with dose-proportional pharmacokinetics. Regression of log-transformed parameters confirmed proportionality, with slopes (90% CI) of 0.884 (0.687–1.081) for ${\text{AUC}}_{0-\infty }$ , 0.893 (0.698–1.089) for AUC_0–tlast_, and 1.001 (0.782–1.220) for C_max_. Considering single-dose administration in the fasted state, geometric mean AUC_0–tlast_ increased from 432.3 h μg/mL (600 mg) to 967.1 h μg/mL (1,500 mg); similar increases were observed for AUC_0–24h_ and ${\text{AUC}}_{0-\infty }$ . Terminal half-life (geometric mean t_1/2_) was consistent across doses (13.9–16.3 h), as was λz (0.043–0.050 h^-1^).

**TABLE 4:** Summary of PK parameters zapnometinib–SAD part.

Parameter	​	600 mg fasted (N = 8)	600 mg fed (N = 8)	900 mg fasted (N = 8)	1,200 mg fasted (N = 8)	1,500 mg fasted (N = 8)
AUC_0–24h_ (h*µg/mL)	GeoMean GeoCV%	287.3 13.0	456.1 14.1	404.6 27.7	558.1 16.8	688.2 24.7
AUC_0–24h_/D (h*ng/ml/mg)	GeoMean GeoCV%	478.8 13.0	760.2 14.1	449.5 27.7	465.1 16.8	458.8 24.7
AUC_0-tlast_ (h*µg/mL)	GeoMean GeoCV%	432.3 20.0	590.6 14.8	652.0 31.6	848.2 17.9	967.1 21.1
AUC_0-tlast_/D (h*ng/ml/mg)	GeoMean GeoCV%	720.5 20.0	984.3 14.8	724.5 31.6	706.8 17.9	644.7 21.1
AUC_0-inf_ (h*µg/mL)	GeoMean GeoCV%	439.8 21.3	596.9 15.5	666.0 31.0	861.4 18.3	974.9 20.7
AUC_0-inf_/D (h*ng/ml/mg)	GeoMean GeoCV%	732.9 21.3	994.8 15.5	740.0 31.0	717.8 18.3	650.0 20.7
C_max_ (µg/mL)	GeoMean GeoCV%	21.0 18.7	52.4 8.9	27.4 32.6	43.5 28.4	50.1 18.7
C_max_/D (ng/mL/mg)	GeoMean GeoCV%	35.0 18.7	87.4 8.9	30.4 32.6	36.3 28.4	33.4 18.7
t_max_ (h)	Min; max Median	2.00; 4.57 4.50	2.00; 6.00 4.50	1.00; 9.00 3.73	2.00; 9.00 4.50	2.03; 12.03 5.00
t_1/2_ (h)	GeoMean GeoCV%	15.83 22.8	15.97 18.0	16.29 22.3	15.46 18.9	13.91 15.6
λ_z_ (/h)	GeoMean GeoCV%	0.044 22.8	0.043 18.0	0.043 22.3	0.045 18.9	0.050 15.6

D = dose; GeoCV% = geometric coefficient of variation in percent; GeoMean = geometric mean; Max =.

maximum; Min = minimum.

#### 3.3.2 MAD pharmacokinetics

PK profiles were also characterized after once-daily dosing over seven consecutive days. The aim was to assess drug accumulation, steady-state trough levels, and dose proportionality following repeated administration of zapnometinib.

**FIGURE 2:**
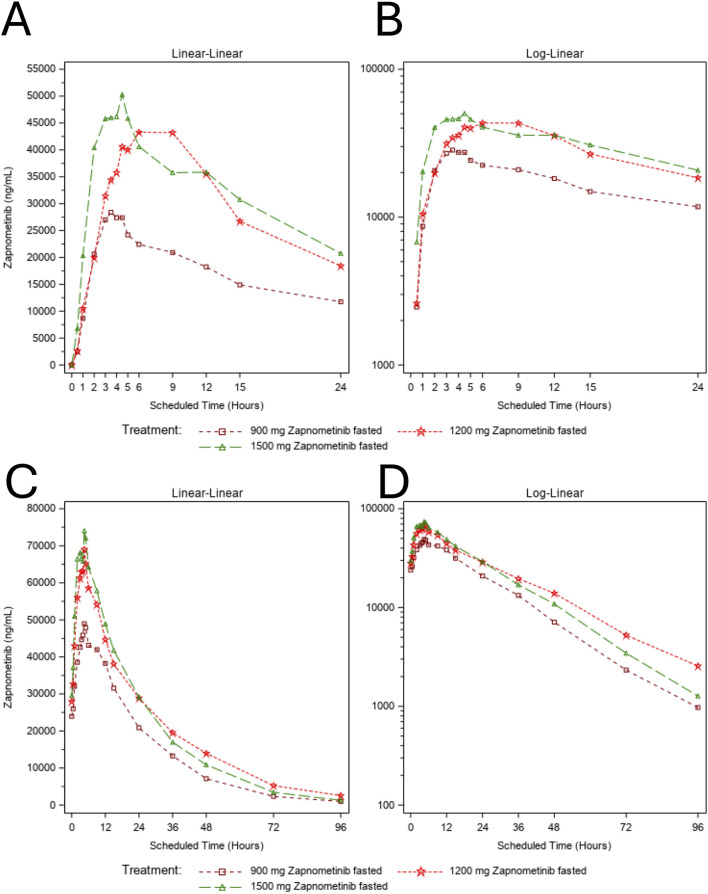
Mean Plasma Concentration–Time Profiles of zapnometinib – MAD Part. Dose Escalation. Plasma concentration–time profiles of zapnometinib following multiple ascending doses (MAD study, once-daily dosing for 7 days). Data represent arithmetic mean plasma concentrations for each dose cohort (900 mg, 1,200 mg, 1,500 mg). **(A,B)** Plasma concentration–time profiles on Day 1 shown on a linear scale **(A)** and a logarithmic scale **(B)**. Concentrations increased with dose, exhibited approximately dose-proportional pharmacokinetics, and remained above the LLOQ (50 ng/mL) throughout the dosing interval. **(C,D)** Arithmetic mean plasma concentration for each dose cohort (900 mg, 1,200 mg, 1,500 mg) on Day 7. Data are presented on a linear scale **(C)** and a logarithmic scale **(D)**.

In the MAD part, plasma concentrations increased dose-dependently on both Day 1 and Day 7 (Figures 2A–D), with peak levels observed at 4.5–6 h post-final dose and reaching up to 74.1 μg/mL at 1,500 mg (Figures 2C,D). Trough concentrations stabilized from Day 5 onwards (Supplementary Figure S2), and drug levels remained quantifiable up to 96 h post last dose (Figures 2C,D). On Day 1, mean peak concentrations were 28.4 μg/mL at 900 mg (3.5 h), 43.3 μg/mL at 1,200 mg (6 h), and 50.3 μg/mL at 1,500 mg (4.5 h), followed by continuous declines in all cohorts. Overall, the pharmacokinetic profile was consistent with dose-proportional exposure. After the first administration, geometric mean AUC_0–24h_ increased from 437.3 h μg/mL (900 mg) to 753.0 h μg/mL (1,500 mg), corresponding to a ∼1.72-fold change with a dose increase of 1.67-fold. Geometric mean C_max_ rose from 28.7 μg/mL (900 mg) to 52.0 μg/mL (1,500 mg), a ∼1.81-fold change consistent with dose proportionality (Table 5). Median T_max_ ranged from 3.5 to 4.5 h, and no relevant differences were observed for geometric mean t_1/2_ (13.5–14.9 h).

**TABLE 5:** Summary of PK parameters zapnometinib–MAD part, day 1.

Parameter	​	900 mg (N = 8)	1,200 mg (N = 8)	1,500 mg (N = 7)
AUC_0–24h_ (h*µg/mL)	n GeoMean GeoCV%	6 437.3 14.5	8 655.0 44.5	7 752.9 24.7
AUC_0–24h_/D (h*ng/ml/mg)	n GeoMean GeoCV%	6 485.9 14.5	8 545.8 44.5	7 501.9 24.7
AUC_0-inf_ (h*µg/mL)	n GeoMean GeoCV%	0^a^ - -	2^a^ 987.7 27.5	0^a^ - -
AUC_0-inf_/D (h*ng/ml/mg)	n GeoMean GeoCV%	0^a^ - -	2^a^ 823.0 27.5	0^a^ - -
C_max_ (µg/mL)	n GeoMean GeoCV%	8 28.7 25.1	8 52.8 45.2	7 52.0 19.6
C_max_/D (ng/mL/mg)	n GeoMean GeoCV%	8 31.9 25.1	8 44.0 45.2	7 35.0 19.6
t_max_ (h)	n Min;Max Median	3.00; 4.50 3.50	8 3.00; 9.00 3.76	7 2.00; 4.52 4.50
t_1/2_ (h)	n GeoMean GeoCV%	6 13.47 30.3	8 14.89 56.5	7 14.07 33.8
λ_z_ (/h)	n GeoMean GeoCV%	6 0.051 30.3	8 0.047 56.5	7 0.049 33.8

D = dose; GeoCV% = geometric coefficient of variation in percent; GeoMean = geometric mean; Max = maximum; Min = minimum.

^a^ Due to *R*2 < 0.8 and/or %AUC_extrap_ > 20%.

AUC_0-inf_ after the first administration could only be determined reliably in 2 subjects.

Arithmetic mean trough concentrations increased comparably across all dose groups until stable levels were reached from Day 5 onwards (Supplementary Figure S2). After administration on Day 7, plasma concentrations rose in a dose-dependent manner, with peak values observed at 4.5 h post-dose in all cohorts: 50.0 μg/mL (900 mg), 69.0 μg/mL (1,200 mg), and 74.1 μg/mL (1,500 mg) (Figures 2C,D). Zapnometinib plasma concentrations declined thereafter but remained above the LLOQ up to 96 h post-dose. Thus, this pharmacokinetic profile was consistent with dose-proportional pharmacokinetics. Geometric mean AUC_0–24h_ increased from 811.1 h μg/mL (900 mg) to 1,080.1 h μg/mL (1,500 mg), corresponding to a ∼1.33-fold change with a 1.67-fold dose increase. Similar results were obtained for AUC_0–tlast_ (Table 6). Geometric mean C_max_ increased from 51.8 μg/mL (900 mg) to 74.2 μg/mL (1,500 mg), a ∼1.43-fold change consistent with dose proportionality. Median T_max_ ranged from 3.5 to 4.5 h, and no relevant differences were observed in geometric mean t_1/2_ (14.8–18.3 h) or λz (0.038–0.047 h^-1^). Moderate accumulation was observed after repeated dosing. The geometric mean accumulation ratios ranged from 1.32 to 1.80 for C_max_ and 1.44 to 1.84 for AUC_0–24h_ across the investigated dose levels (Table 6).

**TABLE 6:** Summary of PK parameters zapnometinib–MAD part, day 7.

Parameter	900 mg (N = 8)		1,200 mg (N = 8)	1,500 mg (N = 7)
AUC_0-tlast_ (h*µg/mL)	GeoMean GeoCV%	127.0 27.3	177.0 40.5	163.0 56.1
AUC_0-tlast_/D (h*ng/ml/mg)	GeoMean GeoCV%	1411.1 27.3	1483.2 40.5	1086.9 56.1
AUC_0–24h_ (h*µg/mL)	GeoMean GeoCV%	811.1 23.1	102.9 34.0	108.0 45.4
AUC_0–24h_/D (h*ng/ml/mg)	GeoMean GeoCV%	901.2 23.1	857.1 34.0	720.1 45.4
C_max_ (µg/mL)	GeoMean GeoCV%	51.8 11.8	69.6 34.0	74.2 33.6
C_max_/D (ng/mL/mg)	GeoMean GeoCV%	57.5 11.8	58.0 34.0	49.4 33.6
t_max_ (h)	Min; max Median	2.00; 12.00 4.53	2.00; 5.00 3.75	2.00; 5.00 3.50
t_1/2_ (h)	GeoMean GeoCV%	15.46 13.8	18.29 21.3	14.75 15.7
λ_z_ (/h)	GeoMean GeoCV%	0.045 13.8	0.038 21.3	0.047 15.7
Accumulation ratio (Day 7/Day 1)				
R_acc_ (C_max_)	​	1.80	1.32	1.43
R_acc_ (AUC_0–24h)_	​	1.84	1.57	1.44

D, dose; GeoCV%, geometric coefficient of variation in percent; GeoMean, geometric mean; Max, maximum; Min, minimum; R_acc_ = accumulation ratio calculated as the ratio of geometric mean exposure parameters at Day 7 versus Day 1.

#### 3.3.3 FDI pharmacokinetics

Because food intake may substantially alter drug absorption and systemic exposure, a dedicated FDI analysis was performed. Zapnometinib pharmacokinetics were compared between fasted and fed conditions to quantify potential differences in C_max_, AUC, and T_max_.

Following administration under fed conditions, median T_max_ was slightly delayed compared with the fasted state (5.0 h vs. 3.7 h). Peak concentrations were substantially higher under fed conditions, with the highest arithmetic mean concentration observed at 4.5 h post-dose (46.7 μg/mL), approximately 2.3-fold higher than in the fasted state (Figures 1C,D). Plasma concentrations declined thereafter but remained higher than under fasted conditions until approximately 15 h post-dose. From 24 h onwards, concentration–time profiles were comparable between conditions, and concentrations remained above the LLOQ at 96 h post-dose in all subjects.

Geometric mean exposure parameters confirmed a food effect. AUC_0–tlast_ increased from 432.3 h μg/mL (600 mg, fasted) to 590.6 h μg/mL (600 mg, fed), corresponding to an approximately 1.4-fold increase, with similar changes observed for AUC_0–24h_ and ${\text{AUC}}_{0-\infty }$ (Table 7). Geometric mean C_max_ increased from 21.0 μg/mL (fasted) to 52.4 μg/mL (fed), representing an approximately 2.5-fold increase. Median T_max_, terminal half-life (t_1/2_), and λz were otherwise comparable between conditions.

**TABLE 7:** Food-drug interaction assessment zapnometinib (600 mg).

Parameter	Treatment	N	Geo LS mean	Ratio (fed/fasted) (%)	90% CI of ratio (%)	Intra CV (%)
AUC_0-tlast_ (h*µg/mL)	Fed	8	590.6	136.61	122.52; 152.31	11.53
	Fasted	8	432.3			
AUC_0-inf_ (h*µg/mL)	Fed	8	596.9	135.73	121.47; 151.67	11.76
	Fasted	8	439.8			
C_max_ (µg/mL)	Fed	8	52.4	249.52	224.15; 277.76	11.36
	Fasted	8	21.0			

CI, confidence interval; fasted = 600 mg, fasted; fed = 600 mg, fed; Intra CV, intrasubject coefficient of variation; Geo LS, Mean = geometric least-squares mean; Ratio (fed/fasted) = geometric least-square mean ratio “fed/fasted”.

#### 3.3.4 DDI pharmacokinetics

Potential DDIs were assessed using probe substrates for CYP2C8 (repaglinide) and CYP2C9 (celecoxib) to evaluate whether zapnometinib alters the pharmacokinetics of co-administered drugs metabolized by these enzymes.

##### 3.3.4.1 Repaglinide (CYP2C8 probe substrate)

Plasma concentrations of repaglinide increased rapidly post-dose, peaking at 0.5 h when administered alone (17.8 nmol/L) and at 0.75 h when co-administered with zapnometinib (16.2 nmol/L). Concentrations then declined steadily but remained above the LLOQ for 12 h under both conditions (Figures 1E,F). Geometric mean AUC_0–tlast_ rose from 25.3 h nmol/L (repaglinide alone) to 35.0 h nmol/L (repaglinide + zapnometinib), representing a ∼1.4-fold increase, with similar changes observed for ${\text{AUC}}_{0-\infty }$ (Table 8). Geometric mean C_max_, median T_max_, t_1/2_, and λz were comparable between treatments. Statistical analysis confirmed this effect: the geometric LS mean ratio (Z + R/R) was 138.3% (90% CI: 126.0–151.7) for AUC_0–tlast_, 139.7% (90% CI: 127.5–153.1) for ${\text{AUC}}_{0-\infty }$ , and 94.5% (90% CI: 82.6–108.2) for C_max_ (Table 9). Ratios for AUC exceeded 100% with 90% CIs outside the acceptance interval, indicating an increase in AUC, consistent with weak CYP2C8 inhibition.

**TABLE 8:** Summary of PK parameters repaglinide (0.5 mg) and celecoxib (100 mg) without/with zapnometinib 1,500 mg/day, each.

Parameter		Repaglinide (+Zapnometinib from day −1 to day 1)		Celecoxib (+Zapnometinib from day −1 to day 3)	
		Day −2 (N = 11)	Day 1 (N = 11)	Day −4 (N = 11)	Day 1 (N = 11)
AUC_0-tlast_ (h*nmol/L)	GeoMean GeoCV%	25.3 36.9	35.0 27.1	9.4 36.0	9.4 41.9
AUC_0-inf_ (h*nmol/L)	GeoMean GeoCV%	25.8 36.3	36.0 26.8	9.7 35.6	9.8 41.3
C_max_ (nmol/L)	GeoMean GeoCV%	17.3 28.3	16.3 29.2	1.0 42.2	5.9 47.3
t_max_ (h)	Min; max Median	0.50; 0.75 0.50	0.50; 0.77 0.52	1.00; 5.00 2.50	2.00; 12.02 3.00
t_1/2_ (h)	GeoMean GeoCV%	2.84 23.0	3.03 42.9	8.26 20.1	11.17 25.2
λ_z_ (/h)	GeoMean GeoCV%	0.244 23.0	0.229 42.9	0.084 20.1	0.062 25.2

GeoCV%, geometric coefficient of variation in percent; GeoMean, geometric mean; Max, maximum; Min, minimum; Day −2: Repaglinide alone; Day 1: Zapnometinib + Repaglinide; Day −4: celecoxib alone; Day 1: Zapnometinib + Celecoxib.

**TABLE 9:** Drug-drug interaction assessment repaglinide (0.5 mg) celecoxib (100 mg) and zapnometinib (1,500 mg/day).

Parameter	Treatment	N	Geo LS mean	Ratio (Z + R/R), or Z + C/C, resp. (%)	90% CI of ratio (%)	Intra CV (%)
AUC_0-tlast_ (h*nmol/L)	Z + R	11	35.0	138.3	126.0; 151.7	12.1
	R	11	25.3			
​	Z + C	11	9.4	100.6	81.1; 124.7	28.5
​	C	11	9.4			
AUC_0-inf_ (h*nmol/L)	Z + R	11	36.0	139.7	127.5; 153.1	11.9
	R	11	25.8			
​	Z + C	11	9.8	101.8	82.6; 125.6	27.7
​	C	11	9.7			
C_max_ (nmol/L)	Z + R	11	16.3	94.5	82.6; 108.2	17.6
	R	11	17.3			
​	Z + C	11	0.6	58.4	44.5; 76.6	36.2
​	C	11	1.0			

CI, confidence interval; Intra CV, intrasubject coefficient of variation; Geo LS Mean, geometric least-squares mean; R, Repaglinide alone; C, Celecoxib alone; Ratio (Z + R/R), geometric least-squares mean ratio “Zapnometinib + Repaglinide/Repaglinide”; Z + R, Zapnometinib + Repaglinide; Ratio (Z + C/C), geometric least-squares mean ratio “Zapnometinib + Celecoxib/Celecoxib”; Z + C, Zapnometinib + Celecoxib.

##### 3.3.4.2 Celecoxib (CYP2C9 probe substrate)

Celecoxib also showed rapid absorption, with peak levels at 2.5 h post-dose (Figures 1G,H). Peak concentrations were higher when administered alone (840.7 nmol/L) compared with co-administration with zapnometinib (568.5 nmol/L). Celecoxib remained quantifiable up to 24–36 h, with most concentrations dropping below the LLOQ (10 nmol/L) by 72 h post-dose. Geometric mean C_max_ decreased from 1,013 nmol/L (alone) to 591.3 nmol/L (with zapnometinib), a ∼0.58-fold change (Table 9). In contrast, geometric mean AUC_0–tlast_ and ${\text{AUC}}_{0-\infty }$ , as well as median T_max_, t_1/2_, and λz, were comparable under both conditions. ANOVA confirmed this pattern: the LS mean ratio (Z + C/C) was 100.6% (90% CI: 81.1–124.9) for AUC_0–tlast_, 101.8% (90% CI: 82.6–125.6) for ${\text{AUC}}_{0-\infty }$ , and 58.4% (90% CI: 44.5–76.6) for C_max_ (Table 9). While AUC values were unaffected, the marked reduction in C_max_ with 90% CIs outside the acceptance interval indicates an inductive effect of zapnometinib on CYP2C9 activity.

Together, these findings demonstrate that zapnometinib interacts with CYP2C8 and CYP2C9 substrates, leading to increased repaglinide exposure and reduced celecoxib C_max_. These results highlight a clinically relevant potential for zapnometinib-mediated DDIs that should be considered when co-administering drugs metabolized by these pathways.

### 3.4 Pharmacodynamics

**FIGURE 3:**
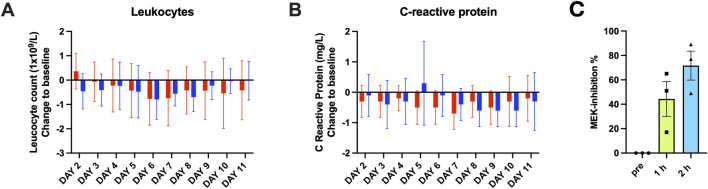
Effects of zapnometinib on leukocyte counts, CRP levels, and ERK phosphorylation in healthy volunteers. **(A)** Leukocyte and **(B)** C-reactive protein (CRP) levels in participants receiving 1,500 mg zapnometinib (blue bars) or placebo (red bars) during the MAD part. Box plots show individual and median values across all time points. No consistent decline was observed, indicating no immunosuppressive or anti-inflammatory effects at this dose. **(C)** Pharmacodynamic effects of zapnometinib on ERK phosphorylation in PBMCs from the SAD 900 mg cohort. Inhibition was evident at 1 h and increased further at 2 h post-dose, consistent with the observed mean T_max_ (3.75 h). Data are depicted as mean values of n = 3 with individual measurements, exploratorily confirming *in vivo* MEK target engagement.

PD effects of zapnometinib were assessed by measuring ERK phosphorylation in PBMCs from participants in the SAD 900 mg cohort. Due to technical limitations of PBMC collection and processing under clinical trial conditions, evaluable samples were available from 3 of 8 participants. Despite the limited sample size, a clear reduction in ERK phosphorylation was observed after dosing compared with baseline (Figure 3C). Inhibition was already evident at 1 h post-dose and was more pronounced at 2 h, which temporally aligns with the T_max_ in this cohort (range 1–9 h; median 3.75 h; Table 1). These data demonstrate *in vivo* MEK target engagement and are consistent with prior clinical observations (Koch-Heier et al., 2022, Figure 4B).

## 4 Discussion

This Phase I study provides a comprehensive clinical characterization of zapnometinib, a selective MEK inhibitor targeting the MAPK/ERK pathway. By integrating safety, tolerability, pharmacokinetic, and pharmacodynamic assessments, as well as analyses of food- and drug–drug interactions, we establish a robust foundation for its further clinical development.

Across the SAD, MAD, and DDI parts of the trial, zapnometinib demonstrated a favorable safety and tolerability profile, with no deaths, serious adverse events, or discontinuations. Reported TEAEs were predominantly mild, transient, and most frequently gastrointestinal or nervous system related, consistent with the known class effects of MEK inhibitors (Mourad et al., 2019; Livingstone et al., 2014). Importantly, neither clinically relevant laboratory abnormalities nor adverse effects on vital signs or ECGs were observed. The additional analysis of leukocyte counts, and CRP levels also indicates that zapnometinib, even at the highest daily dose of 1,500 mg, does not compromise immune cell homeostasis or induce systemic inflammatory markers, and provides no indication of an increased host susceptibility to infections. These results complement previous preclinical and clinical observations, reinforcing the conclusion that zapnometinib does not exert immunosuppressive effects (Rohde et al., 2023; Koch-Heier et al., 2024; Füll et al., 2025).

Pharmacokinetic analyses revealed dose-proportional increases in C_max_ and AUC after both single and repeated administration, with a terminal half-life of ∼14–16 h supporting once-daily dosing. Steady state was achieved within 5 days in the MAD part, with limited accumulation. The FDI study demonstrated that a high-fat meal substantially increased zapnometinib exposure, particularly C_max_, suggesting enhanced absorption under fed conditions. While this effect could be advantageous for maximizing systemic exposure, it also highlights the need to carefully define dosing recommendations to ensure consistency in future clinical trials and therapeutic applications.

The DDI analyses identified measurable interactions with probe substrates of CYP2C8 and CYP2C9. Zapnometinib increased repaglinide exposure, consistent with inhibition of CYP2C8, while co-administration with celecoxib reduced C_max_ without affecting overall exposure, a finding that warrants further investigation regarding potential effects on CYP2C9. Although the magnitude of these effects was moderate, they underscore the importance of considering zapnometinib-mediated DDIs in clinical settings, particularly when combined with drugs that have narrow therapeutic indices (Schreiber et al., 2022a).

Pharmacodynamic assessments confirmed *in vivo* MEK pathway engagement, as evidenced by inhibition of ERK phosphorylation in PBMCs from the SAD 900 mg cohort. Our experience illustrates the challenges of translating the laboratory method of measuring ERK-phosphorylation into the clinical routine. Transport delays occasionally compromised phosphorylation integrity, and when this occurred at baseline, all subsequent samples from that participant had to be excluded. These observations highlight important practical constraints of the trial setting and provide guidance for optimizing sample handling in future studies. Despite technical limitations restricting sample availability, the observed time-dependent inhibition aligns well with plasma concentration–time profiles and with previous findings in clinical studies (Koch-Heier et al., 2022). Inhibition was observed within the first 1–2 h post-dose. While this coincides with the expected T_max_ range, the finding is limited to three evaluable subjects in the 900 mg cohort and should be interpreted cautiously. These results provide direct mechanistic evidence for zapnometinib’s mode of action in humans and further validate its pharmacological rationale.

Taken together, these findings highlight zapnometinib as a promising candidate for indications involving dysregulated MAPK/ERK signaling. The predictable PK, dose-proportionality, and direct evidence of target engagement support its further evaluation in clinical settings. The available data do not suggest immunosuppressive effects of zapnometinib, which is an important consideration in the context of viral infections where both antiviral and immunomodulatory activities are desirable. However, this conclusion is limited, as the present study included only healthy young volunteers, was restricted to 7 days of treatment, and relied only on certain clinical readouts such as leukocyte counts and CRP. More sensitive and disease-relevant biomarkers, as well as longer treatment periods and studies in patient populations, will be required for a more specific examination of potential immunomodulatory or immunosuppressive actions. This point is of particular relevance given the experience with other kinase inhibitors, where unwanted immunosuppressive effects have occasionally been observed (Dennison et al., 2021; De et al., 2024; Wang et al., 2024). The observed food effect and CYP-mediated interactions require careful management in subsequent trials but do not represent treatment-limiting factors.

In conclusion, zapnometinib was safe, well tolerated, and pharmacologically active in healthy volunteers. These data support its continued clinical development, including Phase II trials in viral and inflammatory diseases, where host-directed modulation of the MAPK/ERK pathway offers a novel therapeutic strategy.

## Data Availability

The raw data supporting the conclusions of this article will be made available by the authors, without undue reservation.
