# Supplementary material for: Safety, tolerability, pharmacokinetics, and pharmacodynamics of zapnometinib: results from a phase I clinical study
Source: Front Pharmacol. 2026 Apr 17;17:1743230. doi: 10.3389/fphar.2026.1743230 (PMC13132832; doi:10.3389/fphar.2026.1743230)
Supplement: Supplementary file 1 [file Supplementaryfile1.docx]

Supplementary Material

# Supplementary Data

**S1: Oversight responsibilities and methods, including regulatory compliance and data monitoring processes**

This study was conducted in accordance with the Declaration of Helsinki, ICH Good Clinical Practice (GCP) guidelines, the German Drug Law, and all applicable local regulations. The study protocol, informed consent form, and amendments were reviewed and approved by the responsible Independent Ethics Committee (IEC) prior to initiation. Oversight of study conduct was shared between the sponsor (Atriva Therapeutics GmbH), the principal investigator, and the contracted CRO (Nuvisan GmbH). The sponsor retained ultimate accountability for delegated tasks, including monitoring, data management, and statistical analyses. A detailed Monitoring Plan defined risk-based approaches, including central, remote, and on-site monitoring, as well as processes for addressing noncompliance. Continuous data verification and regular Safety Assessment Meetings between investigator and sponsor ensured subject safety before dose escalation. Data quality and integrity were maintained through source data verification, Good Clinical Data Management practices, and predefined database lock procedures. Audit certificates and documentation of quality assurance procedures are available in the appendices.

**S2: Detailed information regarding the composition, quality control (QC), and manufacturing processes of the drug substance and drug product**

Zapnometinib (ATR-002) is a small-molecule MEK1/2 inhibitor provided as immediate-release film-coated tablets for oral administration. Each active tablet contained 300 mg zapnometinib as drug substance; matching placebo tablets were manufactured to ensure blinding. The investigational medicinal products (IMPs) were produced under Good Manufacturing Practice (GMP) conditions by Rottendorf Pharma GmbH (Germany). Batch numbers used in the study were 7033401C1 (active) and 7033501C1 (placebo), with expiry dates adjusted according to study cohort timelines.

Quality control included release testing of identity, potency, purity, and stability in compliance with ICH and EMA guidelines. Repaglinide (0.5 mg tablets, Holsten Pharma GmbH, Germany) and celecoxib (100 mg capsules, Heumann Pharma, Germany) served as probe substrates in the drug–drug interaction part of the study. All IMPs were packaged and labelled by Nuvisan GmbH in accordance with study-specific and regulatory requirements. Documentation of analytical methods, stability testing, and certification of GMP compliance were archived within the study master file.

**S3: Additional bioanalytical details, including chromatograms, calibration curves, and method validation parameters**

Pharmacokinetic plasma samples were analyzed by Nuvisan GmbH using a validated LC–MS/MS method under GLP and GCP standards. The method was fully validated in accordance with EMA and FDA bioanalytical guidelines and included evaluation of selectivity, sensitivity, linearity, accuracy, precision, stability, and recovery.

Calibration curves for zapnometinib were established over the range of 50–50,000 ng/mL using weighted linear regression (1/x²). Correlation coefficients (R²) consistently exceeded 0.99 across validation runs. Representative chromatograms demonstrated clear peak separation without relevant interference from endogenous matrix components.

Accuracy was within 85–115% of nominal concentrations at low, medium, and high QC levels, while precision (%CV) did not exceed 15% (20% at the LLOQ). The lower limit of quantification (LLOQ) for zapnometinib was 50 ng/mL. Intra-day (n=6 replicates) and inter-day (n=18 replicates across 3 days) variability fulfilled acceptance criteria.

Extraction recovery was consistent, with mean recoveries >85% across QC levels. Matrix effects were assessed in plasma from at least six individual donors and were within acceptable ranges (<15% deviation). Stability testing confirmed analyte integrity under bench-top (6 h), freeze–thaw (3 cycles), and long-term storage conditions (–80 °C for ≥6 months). Processed sample stability was demonstrated for at least 48 h at autosampler conditions (10 °C).

Repaglinide and celecoxib, used in the DDI part of the study, were quantified by analogous validated LC–MS/MS methods, with comparable assay performance. Internal standards (stable isotope–labelled analogs) were employed for all analytes to ensure reliable quantification.

QC samples (low, medium, high, and LLOQ) were interspersed at ≥5% frequency in each analytical batch, and ≥67% of QC results met the predefined acceptance criteria (±15% of nominal, ±20% for LLOQ). These results confirmed robustness and reproducibility of the analytical method throughout the study.

**S4: Additional details on the methodology for summarizing and reporting the PK data, as well as the statistical evaluations**

Pharmacokinetic analyses were performed using validated non-compartmental methods in Phoenix WinNonlin® (Version 7.0 or higher), with additional statistical analyses conducted in SAS® (Version 9.4 or higher). PK parameters included C_max_, t_max_, AUC_0–tlast_, AUC_0–inf_, AUC_0–τ_, t_½_, λz, %AUC_extrap_, and accumulation ratios (R_acc_). C_max_ and t_max_ were derived directly from observed concentration–time data, while AUC values were calculated using the linear/log trapezoidal rule. λz was estimated from the terminal elimination phase using at least three data points; parameters were flagged as unreliable if R² <0.8 or if extrapolated AUC exceeded 20% of total exposure. Plasma concentrations below the LLOQ were set to zero, and missing values were not imputed.

Descriptive statistics (mean, SD, median, minimum, maximum, geometric mean, and %CV) were provided by cohort and study part (SAD, MAD, FDI, and DDI). Dose proportionality was assessed using a power model applied to log-transformed C_max_ and AUC values, with exploratory 90% confidence intervals for slope estimates. Food-drug interaction (FDI) was evaluated by ANOVA including subject and treatment (fed vs. fasted) as factors; least-squares (LS) means and geometric mean ratios with 90% CIs were reported. Drug–drug interactions (DDI) were assessed by analogous ANOVA models comparing probe substrates (repaglinide, celecoxib) with and without zapnometinib. Results were back-transformed to the original scale for interpretation.

No formal statistical hypotheses were defined, as this was an exploratory Phase I trial. All analyses were conducted on the predefined PK analysis set, excluding subjects with major protocol deviations affecting PK data.

**S5: Additional details on PBMC isolation, pERK quantification methods, and data analysis approaches**

Peripheral blood mononuclear cells (PBMCs) were isolated from whole blood samples collected at scheduled timepoints using standard Ficoll^®^ density gradient centrifugation under controlled conditions. Freshly isolated PBMCs were processed promptly to minimize *ex vivo* activation.

Pharmacodynamic assessments were based on quantification of phosphorylated ERK (pERK) relative to total ERK (tERK). Following stimulation with PMA/ionomycin, intracellular pERK and tERK were detected by flow cytometry using phospho-specific antibodies, according to validated procedures. The percentage of phosphorylated ERK was calculated as:

%pERK = (2 × phospho-signal) / (phospho-signal + total signal) × 100.

Baseline-corrected %MEK inhibition was calculated as:

[(%pERKpredose – %pERKbaseline) – (%pERKpostdose – %pERKbaseline)] / (%pERKpredose – %pERKbaseline) × 100,

where %pERKbaseline referred to unstimulated predose controls.

Data were summarized by study part (SAD, MAD, DDI), treatment group, and scheduled sampling times. Results were expressed as descriptive statistics (mean, SD, median, minimum, maximum). Dose- and time-dependent inhibition patterns were analyzed, and graphical summaries were generated to illustrate inhibition kinetics.

Quality control included replicate measurements, internal validation of staining performance, and assessment of inter-assay variability. Assay acceptance criteria were predefined to ensure reproducibility.

# Supplementary Figures

**Supplementary Figure 1:** **Schematic overview of the drug–drug interaction (DDI) study designs**


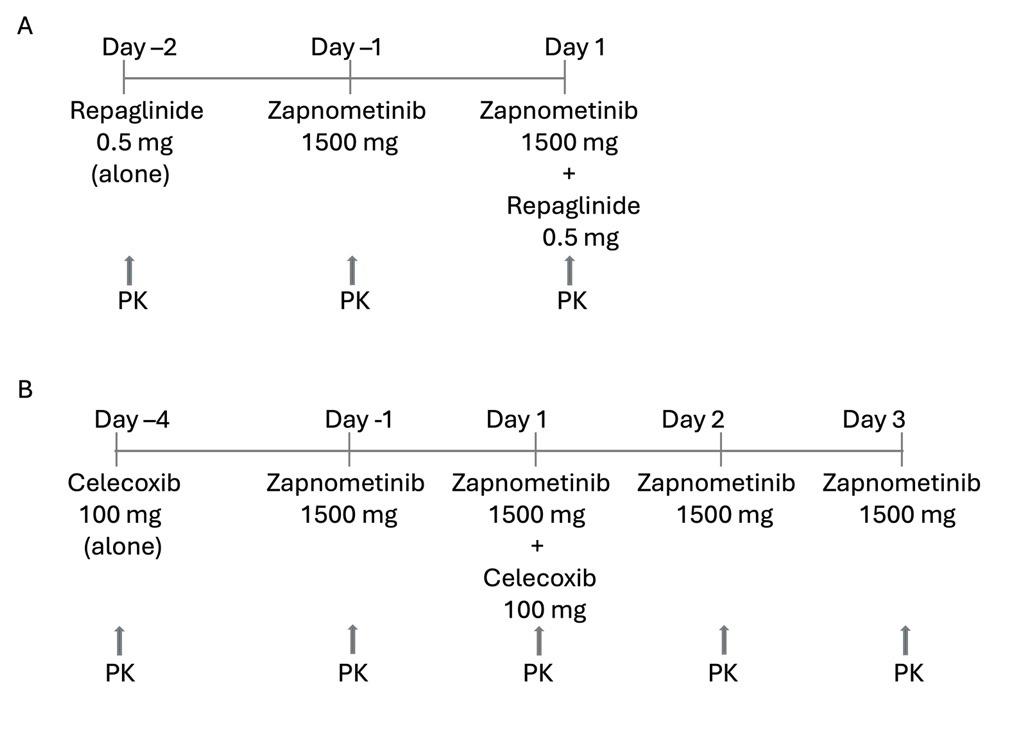


**Figure S1.** (A) Fixed-sequence design evaluating the effect of zapnometinib on CYP2C8 activity using repaglinide as a probe substrate. Repaglinide was administered alone to establish baseline pharmacokinetics, followed by zapnometinib administration with co-administration of repaglinide.
(B) Fixed-sequence design evaluating CYP2C9 activity using celecoxib as a probe substrate. Celecoxib was administered alone, followed by repeated dosing of zapnometinib with co-administration of celecoxib. Pharmacokinetic sampling was performed after probe administration alone and in combination to enable within-subject comparison.

Supplementary Figure 2: Mean C_trough_ Zapnometinib – MAD Part, Day 2 to Day 8


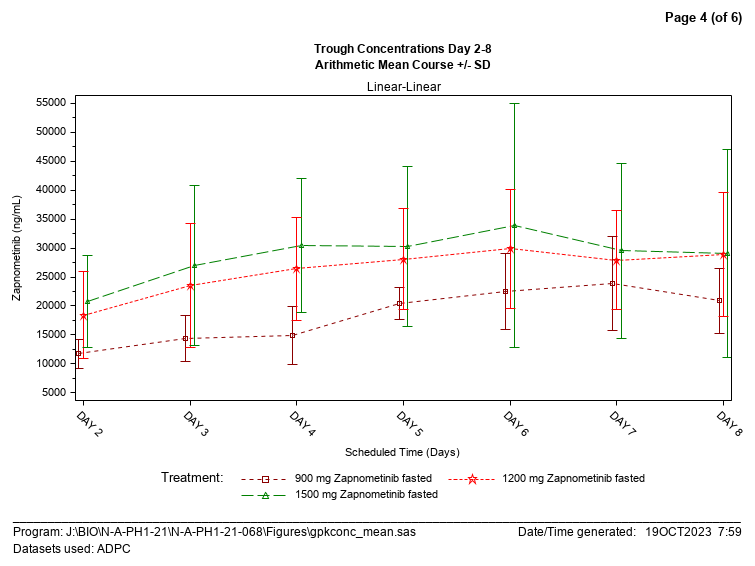


**Figure S2.** Arithmetic mean trough plasma concentrations (±SD) of zapnometinib following once-daily administration of 900, 1200, and 1500 mg under fasted conditions (MAD part, Days 2–8). Trough levels increased over the first treatment days and reached stable concentrations from Day 5 onwards, with quantifiable values maintained through Day 8. Data are shown as arithmetic means with standard deviations.

# Supplementary Tables

**Table S1: Baseline Characteristics of Participants in Phase 1 DDI Study**

| **Parameter** | | **Repaglinide** (N=12) | **Celecoxib** (N=12) |
| --- | --- | --- | --- |
| Age (years) | |  |  |
|  | Mean (SD) | 34.8 (10.90) | 40.9 (10.61) |
|  | Med. (range) | 31.0 (23-53) | 42.5 (25-55) |
| Height (cm) | |  |  |
|  | Mean (SD) | 179.8 (4.91) | 176.6 (10.36) |
|  | Med. (range) | 179.8 (170-187) | 175.0 (161-203) |
| Weight (kg) | |  |  |
|  | Mean (SD) | 81.14 (8.267) | 79.77 (11.958) |
|  | Med. (range) | 80.65 (67.0-96.3) | 75.40 (65.3-111.2) |
| BMI (kg/m^2^) | |  |  |
|  | Mean (SD) | 25.07 (2.014) | 25.51 (2.195) |
|  | Med. (range) | 25.05 (22.0-28.0) | 25.30 (22.5-29.3) |
| Sex | |  |  |
|  | Female | 1 (8.3) | 3 (25.0) |
|  | Male | 11 (91.7) | 9 (75.0) |
| Ethnicity | |  |  |
|  | Not Hispanic or Latino | 12 (100.0) | 12 (100.0) |
| Race | |  |  |
|  | White | 12 (100.0) | 12 (100.0) |

**Table S2: Baseline Characteristics of Participants in Phase 1 MAD Study**

| **Parameter** | | **Placebo** (N=6) | **900 mg** (N=8) | **1,200 mg** (N=8) | **1,500 mg** (N=7) | **Overall** (N=29) |
| --- | --- | --- | --- | --- | --- | --- |
| Age (years) | |  |  |  |  |  |
|  | Mean (SD) | 37.5 (8.38) | 38.3 (10.32) | 40.9 (10.34) | 44.4 (7.09) | 40.3 (9.16) |
|  | Med. (range) | 37.5 (24-46) | 34.0 (25-53) | 39.0 (27-55) | 43.0 (34-54) | 40.0 (24-55) |
| Height (cm) | |  |  |  |  |  |
|  | Mean (SD) | 178.7 (3.78) | 184.1 (5.49) | 176.3 (9.88) | 174.6 (10.69) | 178.5 (8.56) |
|  | Med. (range) | 177.5 (176-186) | 184.5 (177-195) | 177.0 (163-190) | 180.0 (153-183) | 180.0 (153-195) |
| Weight (kg) | |  |  |  |  |  |
|  | Mean (SD) | 78.67 (12.985) | 81.63 (10.331) | 82.45 (9.557) | 79.14 (9.247) | 80.64 (10.028) |
|  | Med. (range) | 76.70 (61.0-98.5) | 78.30 (70.4-97.8) | 85.90 (67.8-92.5) | 75.80 (68.0-93.9) | 79.70 (61.0-98.5) |
| BMI (kg/m^2^) | |  |  |  |  |  |
|  | Mean (SD) | 24.58 (3.380) | 24.15 (3.523) | 26.49 (1.535) | 26.06 (2.968) | 25.34 (2.941) |
|  | Med. (range) | 24.35 (19.7-28.5) | 23.35 (20.2-28.8) | 26.15 (25.1-29.9) | 26.90 (21.8-29.0) | 26.00 (19.7-29.9) |
| Sex | |  |  |  |  |  |
|  | Female | 1 (16.7) | 0 (0.0) | 3 (37.5) | 2 (28.6) | 6 (20.7) |
|  | Male | 5 (83.3) | 8 (100.0) | 5 (62.5) | 5 (71.4) | 23 (79.3) |
| Ethnicity | |  |  |  |  |  |
|  | Not Hispanic or Latino | 6 (100.0) | 8 (100.0) | 8 (100.0) | 7 (100.0) | 29 (100.0) |
| Race | |  |  |  |  |  |
|  | White | 6 (100.0) | 8 (100.0) | 7 (87.5) | 7 100.0) | 28 (96.6) |
|  | Black or African American | 0 (0.0) | 0 (0.0) | 1 (12.5) | 0 (0.0) | 1 (3.4) |

BMI=body mass index; Med=median; SD=standard deviation

**Table S3: TEAEs – SAD Part, Dose Escalation**

| **SOC** | **PT** | **Placebo**  **(N=10)**  **n (%) e** | **600 mg**  **(N=8)**  **n (%) e** | **900 mg**  **(N=8)**  **n (%) e** | **1,200 mg**  **(N=8)**  **n (%) e** | **1,500 mg**  **(N=9)**  **n (%) e** | **Overall**  **(N=43)**  **n (%) e** |
| --- | --- | --- | --- | --- | --- | --- | --- |
| Total |  | 2 (20.0) 4 | 2 (25.0) 3 | 3 (37.5) 4 | 3 (37.5) 4 | 7 (77.8) 13 | 17 (39.5) 28 |
| Gastrointestinal disorders | Total | 0 (0.0) 0 | 0 (0.0) 0 | 1 (12.5) 1 | 3 (37.5) 3 | 3 (33.3) 4 | 7 (16.3) 8 |
|  | Abnormal feces^†^ | - | - | - | - | **2 (22.2) 2** | 2 (4.7) 2 |
|  | Diarrhea^†^ | - | - | - | **2 (25.0) 2** | - | 2 (4.7) 2 |
|  | Nausea | - | - | - | - | **2 (22.2) 2** | 2 (4.7) 2 |
|  | Abdominal discomfort | - | - | - | **1 (12.5) 1** | - | 1 (2.3) 1 |
|  | Vomiting | - | - | **1 (12.5) 1** | - | - | 1 (2.3) 1 |
| Nervous system disorders | Total | 1 (10.0) 1 | 0 (0.0) 0 | 0 (0.0) 0 | 0 (0.0) 0 | 4 (44.4) 6 | 5 (11.6) 7 |
|  | Headache | 1 (10.0) 1 | - | - | - | **2 (22.2) 2** | 3 (7.0) 3 |
|  | Dizziness | - | - | - | - | **2 (22.2) 2** | 2 (4.7) 2 |
|  | Somnolence | - | - | - | - | **1 (11.1) 1** | 1 (2.3) 1 |
|  | Taste disorder | - | - | - | - | **1 (11.1) 1** | 1 (2.3) 1 |
| General disorders and administration site conditions | Total | 0 (0.0) 0 | 1 (12.5) 1 | 1 (12.5) 1 | 1 (12.5) 1 | 1 (11.1) 1 | 4 (9.3) 4 |
|  | Feeling hot | - | - | **1 (12.5) 1** | - | **1 (11.1) 1** | 2 (4.7) 2 |
|  | Catheter site pain | - | - | - | 1 (12.5) 1 | - | 1 (2.3) 1 |
|  | Fatigue | - | **1 (12.5) 1** | - | - | - | 1 (2.3) 1 |
| Skin and subcutaneous tissue disorders | Total | **1 (10.0) 1** | 0 (0.0) 0 | **2 (25.0) 2** | 0 (0.0) 0 | 0 (0.0) 0 | 3 (7.0) 3 |
|  | Eczema | - | - | 1 (12.5) 1 | - | - | 1 (2.3) 1 |
|  | Pruritus | **1 (10.0) 1** | - | - | - | - | 1 (2.3) 1 |
|  | Rash | - | - | **1 (12.5) 1** | - | - | 1 (2.3) 1 |
| Blood and lymphatic system disorders | Total | 1 (10.0) 1 | 0 (0.0) 0 | 0 (0.0) 0 | 0 (0.0) 0 | 0 (0.0) 0 | 1 (2.3) 1 |
|  | Lymphadenopathy | 1 (10.0) 1 | - | - | - | - | 1 (2.3) 1 |
| Infections and infestations | Total | 0 (0.0) 0 | 0 (0.0) 0 | 0 (0.0) 0 | 0 (0.0) 0 | 1 (11.1) 1 | 1 (2.3) 1 |
|  | Oral herpes | - | - | - | - | **1 (11.1) 1** | 1 (2.3) 1 |
| Investigations | Total | 0 (0.0) 0 | 1 (12.5) 2 | 0 (0.0) 0 | 0 (0.0) 0 | 0 (0.0) 0 | 1 (2.3) 2 |
|  | Amylase increased | - | 1 (12.5) 1 | - | - | - | 1 (2.3) 1 |
|  | Lipase increased | - | 1 (12.5) 1 | - | - | - | 1 (2.3) 1 |
| Psychiatric disorders | Total | 1 (10.0) 1 | 0 (0.0) 0 | 0 (0.0) 0 | 0 (0.0) 0 | 0 (0.0) 0 | 1 (2.3) 1 |
|  | Sleep disorder | **1 (10.0) 1** | - | - | - | - | 1 (2.3) 1 |
| Renal and urinary disorders | Total | 0 (0.0) 0 | 0 (0.0) 0 | 0 (0.0) 0 | 0 (0.0) 0 | 1 (11.1) 1 | 1 (2.3) 1 |
|  | Dysuria | - | - | - | - | **1 (11.1) 1** | 1 (2.3) 1 |

e=number of events; n=number of subjects having the event; PT=Preferred Term; SOC=System Organ Class

One (1) subject may have more than 1 event. Numbers printed in **bold** denote drug-related events, i.e., events with a relationship rated as ‘possible’, ‘probable’ or ‘related’. Events coded using MedDRA Version 25.1 (terms adapted to American English spelling in table).

^†^: Diarrhea=More than 5 defecations per day; below that number and abnormal consistency, the term “abnormal feces” was used.

Table S4: Overall Summary of TEAEs – SAD Part, Food-Drug Interaction

|  | **Placebo**  **Fasted**  **(N=2)** | **Placebo**  **Fed**  **(N=2)** | **600 mg**  **Fasted**  **(N=8)** | **600 mg**  **Fed**  **(N=8)** | **Overall**  **(N=10)** |
| --- | --- | --- | --- | --- | --- |
| Subjects with any TEAE | 0 (0.0%) | 1 (50.0%) | 2 (25.0%) | 1 (12.5%) | 4 (40.0%) |
| Subjects with any drug‑related TEAE | 0 (0.0%) | 1 (50.0%) | 1 (12.5%) | 0 (0.0%) | 2 (20.0%) |
| Subjects with any mild TEAE | 0 (0.0%) | 1 (50.0%) | 2 (25.0%) | 1 (12.5%) | 4 (40.0%) |
| Subjects with any serious TEAE | 0 (0.0%) | 0 (0.0%) | 0 (0.0%) | 0 (0.0%) | 0 (0.0%) |
| Subjects with any TEAE leading to death | 0 (0.0%) | 0 (0.0%) | 0 (0.0%) | 0 (0.0%) | 0 (0.0%) |
| Subjects discontinued due to TEAE | 0 (0.0%) | 0 (0.0%) | 0 (0.0%) | 0 (0.0%) | 0 (0.0%) |
| Any TEAE | 0 | 2 | 3 | 1 | 6 |
| Drug‑related TEAE | 0 | 2 | 1 | 0 | 3 |
| Mild TEAE | 0 | 2 | 3 | 1 | 6 |
| Serious TEAE | 0 | 0 | 0 | 0 | 0 |
| TEAE leading to death | 0 | 0 | 0 | 0 | 0 |

TEAE=treatment‑emergent adverse event

Drug‑related TEAE: relationship rated as ‘possible’, ‘probable’ or ‘related’.

Table S5: Overall Summary of TEAEs – Repaglinide Cohort

|  | **Zapnometinib**  **(N=11)** | **Repaglinide**  **(N=12)** | **Zapnometinib + Repaglinide**  **(N=11)** | **Overall**  **(N=12)** |
| --- | --- | --- | --- | --- |
| Subjects with any TEAE | 3 (27.3%) | 0 (0.0%) | 6 (54.5%) | 6 (50.0%) |
| Subjects with any drug‑related TEAE | 3 (27.3%) | 0 (0.0%) | 5 (45.5%) | 5 (41.7%) |
| Subjects with any mild TEAE | 3 (27.3%) | 0 (0.0%) | 5 (45.5%) | 5 (41.7%) |
| Subjects with any moderate TEAE | 0 (0.0%) | 0 (0.0%) | 2 (18.2%) | 2 (16.7%) |
| Subjects with any serious TEAE | 0 (0.0%) | 0 (0.0%) | 0 (0.0%) | 0 (0.0%) |
| Subjects with any TEAE leading to death | 0 (0.0%) | 0 (0.0%) | 0 (0.0%) | 0 (0.0%) |
| Subjects discontinued due to TEAE | 0 (0.0%) | 0 (0.0%) | 0 (0.0%) | 0 (0.0%) |
| Any TEAE | 5 | 0 | 13 | 18 |
| Drug‑related TEAE | 5 | 0 | 11 | 16 |
| Mild TEAE | 5 | 0 | 11 | 16 |
| Moderate TEAE | 0 | 0 | 2 | 2 |
| Serious TEAE | 0 | 0 | 0 | 0 |
| TEAE leading to death | 0 | 0 | 0 | 0 |

TEAE=treatment‑emergent adverse event

Drug‑related TEAE: relationship rated as ‘possible’, ‘probable’ or ‘related’.

Table S6: Overall Summary of TEAEs – Celecoxib Cohort

|  | **Zapnometinib**  **(N=12)** | **Celecoxib**  **(N=12)** | **Zapnometinib + Celecoxib**  **(N=11)** | **Overall**  **(N=12)** |
| --- | --- | --- | --- | --- |
| Subjects with any TEAE | 9 (75.0%) | 3 (25.0%) | 9 (81.8%) | 11 (91.7%) |
| Subjects with any drug‑related TEAE | 9 (75.0%) | 0 (0.0%) | 9 (81.8%) | 11 (91.7%) |
| Subjects with any mild TEAE | 9 (75.0%) | 1 (8.3%) | 9 (81.8%) | 11 (91.7%) |
| Subjects with any moderate TEAE | 1 (8.3%) | 2 (16.7%) | 1 (9.1%) | 4 (33.3%) |
| Subjects with any serious TEAE | 0 (0.0%) | 0 (0.0%) | 0 (0.0%) | 0 (0.0%) |
| Subjects with any TEAE leading to death | 0 (0.0%) | 0 (0.0%) | 0 (0.0%) | 0 (0.0%) |
| Subjects discontinued due to TEAE | 0 (0.0%) | 0 (0.0%) | 0 (0.0%) | 0 (0.0%) |
| Any TEAE | 18 | 3 | 28 | 49 |
| Drug‑related TEAE | 17 | 0 | 26 | 43 |
| Mild TEAE | 17 | 1 | 27 | 45 |
| Moderate TEAE | 1 | 2 | 1 | 4 |
| Serious TEAE | 0 | 0 | 0 | 0 |
| TEAE leading to death | 0 | 0 | 0 | 0 |

TEAE=treatment‑emergent adverse event

Drug‑related TEAE: relationship rated as ‘possible’, ‘probable’ or ‘related’.

**Table S7: TEAEs – MAD Part**

| **SOC** | **PT** | **Placebo**  **(N=6)**  **n (%) e** | **900 mg**  **(N=8)**  **n (%) e** | **1,200 mg**  **(N=8)**  **n (%) e** | **1,500 mg**  **(N=7)**  **n (%) e** | **Overall**  **(N=29)**  **n (%) e** |
| --- | --- | --- | --- | --- | --- | --- |
| Total |  | 2 (33.3) 3 | 5 (62.5) 12 | 7 (87.5) 27 | 3 (42.9) 12 | 17 (58.6) 54 |
| Gastrointestinal disorders | Total | 1 (16.7) 1 | 3 (37.5) 7 | 4 (50.0) 15 | 2 (28.6) 7 | 10 (34.5) 30 |
|  | Abnormal feces^†^ | 1 (16.7) 1 | **2 (25.0) 4** | **4 (50.0) 7** | **1 (14.3) 1** | 8 (27.6) 13 |
|  | Abdominal pain | - | **2 (25.0) 2** | **1 (12.5) 1** | **1 (14.3) 2** | 4 (13.8) 5 |
|  | Abdominal discomfort | - | - | **2 (25.0) 5** | **1 (14.3) 1** | 3 (10.3) 6 |
|  | Abdominal pain upper | - | - | **1 (12.5) 1** | **1 (14.3) 1** | 2 (6.9) 2 |
|  | Nausea | - | - | **1 (12.5) 1** | **1 (14.3) 1** | 2 (6.9) 2 |
|  | Aphthous ulcer | - | **1 (12.5) 1** | - | - | 1 (3.4) 1 |
|  | Toothache | - | - | - | **1 (14.3) 1** | 1 (3.4) 1 |
| Renal and urinary disorders | Total | 0 (0.0) 0 | 0 (0.0) 0 | 4 (50.0) 6 | 1 (14.3) 1 | 5 (17.2) 7 |
|  | Urinary tract pain | - | - | **4 (50.0) 6** | - | 4 (13.8) 6 |
|  | Dysuria | - | - | - | **1 (14.3) 1** | 1 (3.4) 1 |
| Nervous system disorders | Total | 0 (0.0) 0 | 1 (12.5) 1 | 2 (25.0) 2 | 1 (14.3) 3 | 4 (13.8) 6 |
|  | Headache | - | - | **2 (25.0) 2^‡^** | **1 (14.3) 2** | 3 (10.3) 4 |
|  | Dizziness | - | **1 (12.5) 1** | - | **1 (14.3) 1** | 2 (6.9) 2 |
| Skin and subcutaneous tissue disorders | Total | 0 (0.0) 0 | 1 (12.5) 1 | 2 (25.0) 2 | 0 (0.0) 0 | 3 (10.3) 3 |
|  | Acne | - | - | **1 (12.5) 1** | - | 1 (3.4) 1 |
|  | Sebaceous gland disorder | - | - | **1 (12.5) 1** | - | 1 (3.4) 1 |
|  | Seborrheic dermatitis | - | **1 (12.5) 1** | - | - | 1 (3.4) 1 |
| General disorders and administration site conditions | Total | 0 (0.0) 0 | 2 (25.0) 2 | 0 (0.0) 0 | 0 (0.0) 0 | 2 (6.9) 2 |
|  | Pain | - | **2 (25.0) 2** | - | - | 2 (6.9) 2 |
| Infections and infestations | Total | 1 (16.7) 1 | 0 (0.0) 0 | 1 (12.5) 2 | 0 (0.0) 0 | 2 (6.9) 3 |
|  | Cystitis | - | - | **1 (12.5) 1** | - | 1 (3.4) 1 |
|  | Hordeolum | 1 (16.7) 1 | - | - | - | 1 (3.4) 1 |
|  | Oral herpes | - | - | **1 (12.5) 1** | - | 1 (3.4) 1 |
| Investigations | Total | 1 (16.7) 1 | 1 (12.5) 1 | 0 (0.0) 0 | 0 (0.0) 0 | 2 (6.9) 2 |
|  | Lipase increased | - | **1 (12.5) 1** | - | - | 1 (3.4) 1 |
|  | Occult blood | 1 (16.7) 1 | - | - | - | 1 (3.4) 1 |
| Respiratory, thoracic and mediastinal disorders | Total | 0 (0.0) 0 | 0 (0.0) 0 | 0 (0.0) 0 | 1 (14.3) 1 | 1 (3.4) 1 |
|  | Oropharyngeal pain | - | - | - | 1 (14.3) 1 | 1 (3.4) 1 |

**Table S8: Leucocyte count**

|  | | **Placebo**  **fasted (N=6)** | | **900 mg Zapnometinib**  **fasted (N=8)** | | **1,200 mg Zapnometinib**  **fasted (N=8)** | | **1,500 mg Zapnometinib**  **fasted (N=7)** | |
| --- | --- | --- | --- | --- | --- | --- | --- | --- | --- |
| Scheduled  Study Time | Statistics | Absolute | Change from Baseline | Absolute | Change from Baseline | Absolute | Change from Baseline | Absolute | Change from Baseline |
| DAY -1 | n | 6 |  | 8 |  | 8 |  | 7 |  |
|  | Mean | 6.198 |  | 6.091 |  | 6.138 |  | 6.451 |  |
|  | SD | 1.0515 |  | 1.6705 |  | 1.3949 |  | 2.9465 |  |
|  | CV% | 17.0 |  | 27.4 |  | 22.7 |  | 45.7 |  |
|  | Minimum | 4.83 |  | 3.87 |  | 4.24 |  | 4.43 |  |
|  | Median | 6.445 |  | 6.140 |  | 5.780 |  | 5.670 |  |
|  | Maximum | 7.40 |  | 8.93 |  | 8.37 |  | 12.97 |  |
| DAY 1 | n | 6 |  | 8 |  | 8 |  | 7 |  |
|  | Mean | 5.690 |  | 5.244 |  | 5.116 |  | 5.489 |  |
|  | SD | 1.1943 |  | 1.3046 |  | 1.2698 |  | 0.8690 |  |
|  | CV% | 21.0 |  | 24.9 |  | 24.8 |  | 15.8 |  |
|  | Minimum | 4.32 |  | 3.42 |  | 3.66 |  | 4.71 |  |
|  | Median | 5.315 |  | 5.335 |  | 5.085 |  | 4.970 |  |
|  | Maximum | 7.75 |  | 7.15 |  | 6.72 |  | 6.71 |  |
| DAY 2 | n | 6 | 6 | 8 | 8 | 8 | 8 | 7 | 7 |
|  | Mean | 6.055 | 0.365 | 5.473 | 0.229 | 5.204 | 0.088 | 5.027 | -0.461 |
|  | SD | 1.5723 | 0.7283 | 1.2800 | 0.4254 | 1.0976 | 0.5424 | 0.7054 | 0.7258 |
|  | CV% | 26.0 | 199.5 | 23.4 | 186.0 | 21.1 | 619.9 | 14.0 | -157.3 |
|  | Minimum | 4.65 | -0.63 | 3.56 | -0.30 | 3.68 | -0.40 | 4.28 | -1.47 |
|  | Median | 5.665 | 0.660 | 5.920 | 0.145 | 5.210 | -0.055 | 4.820 | -0.370 |
|  | Maximum | 8.63 | 1.09 | 6.85 | 1.02 | 6.86 | 1.13 | 6.34 | 0.60 |
| DAY 3 | n | 6 | 6 | 8 | 8 | 8 | 8 | 7 | 7 |
|  | Mean | 5.625 | -0.065 | 5.539 | 0.295 | 5.331 | 0.215 | 5.080 | -0.409 |
|  | SD | 1.0791 | 0.8105 | 1.2742 | 0.3047 | 1.1156 | 0.7418 | 0.7245 | 0.6555 |
|  | CV% | 19.2 | -1246.9 | 23.0 | 103.3 | 20.9 | 345.0 | 14.3 | -160.4 |
|  | Minimum | 4.32 | -1.14 | 3.44 | -0.11 | 3.31 | -0.83 | 4.51 | -1.49 |
|  | Median | 5.635 | 0.025 | 5.635 | 0.270 | 5.650 | 0.255 | 4.730 | -0.300 |
|  | Maximum | 6.90 | 0.92 | 7.33 | 0.89 | 6.56 | 1.18 | 6.56 | 0.52 |
| DAY 4 | n | 6 | 6 | 8 | 8 | 8 | 8 | 7 | 7 |
|  | Mean | 5.468 | -0.222 | 5.635 | 0.391 | 5.145 | 0.029 | 5.254 | -0.234 |
|  | SD | 1.0281 | 1.0876 | 1.7627 | 0.5794 | 0.9585 | 0.5745 | 1.2510 | 0.9747 |
|  | CV% | 18.8 | -490.7 | 31.3 | 148.1 | 18.6 | 1998.4 | 23.8 | -416.0 |
|  | Minimum | 4.44 | -2.15 | 3.14 | -0.28 | 3.66 | -0.69 | 4.01 | -1.37 |
|  | Median | 5.265 | -0.035 | 5.600 | 0.180 | 5.205 | -0.015 | 4.480 | -0.490 |
|  | Maximum | 7.32 | 1.00 | 8.61 | 1.46 | 6.25 | 0.76 | 7.21 | 1.57 |
| DAY 5 | n | 6 | 6 | 8 | 8 | 8 | 8 | 7 | 7 |
|  | Mean | 5.262 | -0.428 | 5.589 | 0.345 | 5.358 | 0.241 | 5.011 | -0.477 |
|  | SD | 0.7819 | 1.1101 | 1.4515 | 0.8626 | 0.8511 | 0.5439 | 0.9147 | 1.0754 |
|  | CV% | 14.9 | -259.2 | 26.0 | 250.0 | 15.9 | 225.4 | 18.3 | -225.4 |
|  | Minimum | 4.47 | -2.55 | 3.24 | -0.28 | 4.05 | -0.61 | 4.12 | -2.29 |
|  | Median | 5.160 | -0.010 | 5.515 | 0.035 | 5.250 | 0.170 | 4.660 | -0.310 |
|  | Maximum | 6.66 | 0.34 | 7.38 | 2.33 | 6.43 | 1.11 | 6.41 | 1.33 |
| DAY 6 | n | 6 | 6 | 8 | 8 | 8 | 8 | 7 | 7 |
|  | Mean | 4.918 | -0.772 | 5.184 | -0.060 | 4.953 | -0.164 | 4.696 | -0.793 |
|  | SD | 0.9111 | 1.0771 | 0.9799 | 0.4701 | 1.1346 | 0.3507 | 0.7799 | 0.8156 |
|  | CV% | 18.5 | -139.6 | 18.9 | -783.6 | 22.9 | -214.1 | 16.6 | -102.9 |
|  | Minimum | 3.95 | -2.56 | 3.65 | -0.89 | 3.39 | -0.80 | 4.10 | -2.40 |
|  | Median | 4.850 | -0.610 | 5.330 | -0.105 | 5.235 | -0.195 | 4.480 | -0.610 |
|  | Maximum | 6.46 | 0.19 | 6.26 | 0.52 | 6.17 | 0.27 | 6.30 | 0.19 |
| DAY 7 | n | 6 | 6 | 8 | 8 | 8 | 8 | 7 | 7 |
|  | Mean | 4.948 | -0.742 | 5.279 | 0.035 | 5.160 | 0.044 | 4.930 | -0.559 |
|  | SD | 0.7247 | 1.1311 | 1.2734 | 0.4204 | 1.0831 | 0.3905 | 0.7851 | 0.5030 |
|  | CV% | 14.6 | -152.5 | 24.1 | 1201.1 | 21.0 | 892.6 | 15.9 | -90.0 |
|  | Minimum | 4.12 | -2.91 | 3.62 | -0.46 | 3.78 | -0.52 | 3.99 | -1.24 |
|  | Median | 4.760 | -0.340 | 5.250 | 0.115 | 5.170 | 0.120 | 5.270 | -0.620 |
|  | Maximum | 6.08 | 0.27 | 7.11 | 0.73 | 6.68 | 0.48 | 6.09 | 0.42 |
| DAY 8 | n | 6 | 6 | 8 | 8 | 8 | 8 | 7 | 7 |
|  | Mean | 5.265 | -0.425 | 5.166 | -0.078 | 5.059 | -0.058 | 4.791 | -0.697 |
|  | SD | 0.6718 | 0.9676 | 1.1724 | 0.4746 | 1.0168 | 0.4832 | 0.8425 | 0.5972 |
|  | CV% | 12.8 | -227.7 | 22.7 | -612.4 | 20.1 | -840.3 | 17.6 | -85.7 |
|  | Minimum | 4.42 | -2.24 | 3.44 | -0.85 | 3.82 | -1.09 | 3.86 | -1.59 |
|  | Median | 5.200 | -0.190 | 5.445 | -0.010 | 4.880 | 0.055 | 4.890 | -0.590 |
|  | Maximum | 6.17 | 0.56 | 6.78 | 0.40 | 6.58 | 0.53 | 6.27 | 0.02 |
| DAY 9 | n | 6 | 6 | 8 | 8 | 8 | 8 | 7 | 7 |
|  | Mean | 5.257 | -0.433 | 5.683 | 0.439 | 5.490 | 0.374 | 5.263 | -0.226 |
|  | SD | 0.5107 | 1.1797 | 1.2457 | 0.5181 | 0.9173 | 0.7479 | 0.8112 | 0.5713 |
|  | CV% | 9.7 | -272.2 | 21.9 | 118.1 | 16.7 | 200.1 | 15.4 | -253.1 |
|  | Minimum | 4.69 | -2.60 | 3.88 | -0.04 | 3.99 | -1.05 | 4.08 | -1.18 |
|  | Median | 5.140 | -0.220 | 5.755 | 0.250 | 5.610 | 0.435 | 5.430 | -0.130 |
|  | Maximum | 5.88 | 0.63 | 7.42 | 1.61 | 6.52 | 1.62 | 6.63 | 0.58 |
| DAY 10 | n | 6 | 6 | 8 | 8 | 8 | 8 | 7 | 7 |
|  | Mean | 5.145 | -0.545 | 5.655 | 0.411 | 5.346 | 0.230 | 5.450 | -0.039 |
|  | SD | 0.8195 | 1.4424 | 1.2691 | 0.4022 | 0.9995 | 0.4627 | 1.0674 | 0.5047 |
|  | CV% | 15.9 | -264.7 | 22.4 | 97.8 | 18.7 | 201.2 | 19.6 | -1308.5 |
|  | Minimum | 4.10 | -3.07 | 3.54 | 0.05 | 3.92 | -0.66 | 4.25 | -0.58 |
|  | Median | 5.205 | -0.120 | 6.030 | 0.295 | 5.300 | 0.220 | 5.160 | -0.020 |
|  | Maximum | 5.96 | 0.84 | 7.25 | 1.26 | 6.74 | 0.81 | 7.00 | 0.80 |
| DAY 11 | n | 6 | 6 | 8 | 8 | 8 | 8 | 7 | 7 |
|  | Mean | 5.272 | -0.418 | 6.119 | 0.875 | 5.485 | 0.369 | 5.464 | -0.024 |
|  | SD | 0.6579 | 1.2114 | 2.2527 | 1.3372 | 0.9897 | 0.5051 | 1.0937 | 0.7890 |
|  | CV% | 12.5 | -289.6 | 36.8 | 152.8 | 18.0 | 137.0 | 20.0 | -3248.8 |
|  | Minimum | 4.52 | -2.56 | 4.06 | -0.01 | 3.97 | -0.37 | 4.14 | -0.91 |
|  | Median | 5.055 | -0.230 | 5.630 | 0.575 | 5.465 | 0.275 | 5.490 | -0.310 |
|  | Maximum | 6.09 | 0.84 | 11.21 | 4.06 | 7.01 | 1.29 | 7.47 | 1.06 |

**Table S9: C Reactive Protein (mg/L)**

|  | | **Placebo**  **fasted (N=6)** | | **900 mg Zapnometinib**  **fasted (N=8)** | | **1,200 mg Zapnometinib**  **fasted (N=8)** | | **1,500 mg Zapnometinib**  **fasted (N=7)** | |
| --- | --- | --- | --- | --- | --- | --- | --- | --- | --- |
| Scheduled  Study Time | Statistics | Absolute | Change from Baseline | Absolute | Change from Baseline | Absolute | Change from Baseline | Absolute | Change from Baseline |
| DAY -1 | n | 6 |  | 8 |  | 8 |  | 7 |  |
|  | Mean | 0.8 |  | 0.3 |  | 2.6 |  | 1.0 |  |
|  | SD | 0.75 |  | 0.71 |  | 2.88 |  | 0.82 |  |
|  | CV% | 90.3 |  | 282.8 |  | 109.5 |  | 81.6 |  |
|  | Minimum | 0 |  | 0 |  | 0 |  | 0 |  |
|  | Median | 1.0 |  | 0.0 |  | 1.5 |  | 1.0 |  |
|  | Maximum | 2 |  | 2 |  | 9 |  | 2 |  |
| DAY 1 | n | 6 |  | 8 |  | 8 |  | 7 |  |
|  | Mean | 1.0 |  | 0.1 |  | 2.3 |  | 1.0 |  |
|  | SD | 0.63 |  | 0.35 |  | 2.31 |  | 0.82 |  |
|  | CV% | 63.2 |  | 282.8 |  | 102.9 |  | 81.6 |  |
|  | Minimum | 0 |  | 0 |  | 0 |  | 0 |  |
|  | Median | 1.0 |  | 0.0 |  | 1.0 |  | 1.0 |  |
|  | Maximum | 2 |  | 1 |  | 7 |  | 2 |  |
| DAY 2 | n | 6 | 6 | 8 | 8 | 8 | 8 | 7 | 7 |
|  | Mean | 0.7 | -0.3 | 0.1 | 0.0 | 1.9 | -0.4 | 0.9 | -0.1 |
|  | SD | 0.52 | 0.52 | 0.35 | 0.00 | 2.17 | 0.74 | 0.69 | 0.69 |
|  | CV% | 77.5 | -154.9 | 282.8 | 0 | 115.6 | -198.4 | 80.5 | -483.0 |
|  | Minimum | 0 | -1 | 0 | 0 | 0 | -2 | 0 | -1 |
|  | Median | 1.0 | 0.0 | 0.0 | 0.0 | 1.0 | 0.0 | 1.0 | 0.0 |
|  | Maximum | 1 | 0 | 1 | 0 | 7 | 0 | 2 | 1 |
| DAY 3 | n | 6 | 6 | 8 | 8 | 8 | 8 | 7 | 7 |
|  | Mean | 0.7 | -0.3 | 0.1 | 0.0 | 1.8 | -0.5 | 0.6 | -0.4 |
|  | SD | 0.52 | 0.52 | 0.35 | 0.00 | 1.83 | 0.76 | 0.53 | 0.79 |
|  | CV% | 77.5 | -154.9 | 282.8 | 0 | 104.7 | -151.2 | 93.5 | -183.6 |
|  | Minimum | 0 | -1 | 0 | 0 | 0 | -2 | 0 | -1 |
|  | Median | 1.0 | 0.0 | 0.0 | 0.0 | 1.0 | 0.0 | 1.0 | -1.0 |
|  | Maximum | 1 | 0 | 1 | 0 | 6 | 0 | 1 | 1 |
| DAY 4 | n | 6 | 6 | 8 | 8 | 8 | 8 | 7 | 7 |
|  | Mean | 0.8 | -0.2 | 0.1 | 0.0 | 1.4 | -0.9 | 0.7 | -0.3 |
|  | SD | 0.75 | 0.41 | 0.35 | 0.00 | 2.00 | 0.99 | 0.49 | 0.76 |
|  | CV% | 90.3 | -244.9 | 282.8 | - | 145.1 | -113.3 | 68.3 | -264.6 |
|  | Minimum | 0 | -1 | 0 | 0 | 0 | -3 | 0 | -1 |
|  | Median | 1.0 | 0.0 | 0.0 | 0.0 | 1.0 | -1.0 | 1.0 | 0.0 |
|  | Maximum | 2 | 0 | 1 | 0 | 6 | 0 | 1 | 1 |
| DAY 5 | n | 6 | 6 | 8 | 8 | 8 | 8 | 7 | 7 |
|  | Mean | 0.5 | -0.5 | 0.5 | 0.4 | 1.3 | -1.0 | 1.3 | 0.3 |
|  | SD | 0.84 | 0.55 | 1.07 | 1.06 | 1.67 | 1.07 | 1.38 | 1.38 |
|  | CV% | 167.3 | -109.5 | 213.8 | 282.8 | 133.5 | -106.9 | 107.3 | 483.0 |
|  | Minimum | 0 | -1 | 0 | 0 | 0 | -3 | 0 | -1 |
|  | Median | 0.0 | -0.5 | 0.0 | 0.0 | 1.0 | -1.0 | 1.0 | 0.0 |
|  | Maximum | 2 | 0 | 3 | 3 | 5 | 0 | 4 | 3 |
| DAY 6 | n | 6 | 6 | 8 | 8 | 8 | 8 | 7 | 7 |
|  | Mean | 0.5 | -0.5 | 0.4 | 0.3 | 1.4 | -0.9 | 0.9 | -0.1 |
|  | SD | 0.84 | 0.55 | 0.74 | 0.71 | 2.00 | 0.99 | 0.90 | 0.69 |
|  | CV% | 167.3 | -109.5 | 198.4 | 282.8 | 145.1 | -113.3 | 105.0 | -483.0 |
|  | Minimum | 0 | -1 | 0 | 0 | 0 | -3 | 0 | -1 |
|  | Median | 0.0 | -0.5 | 0.0 | 0.0 | 1.0 | -1.0 | 1.0 | 0.0 |
|  | Maximum | 2 | 0 | 2 | 2 | 6 | 0 | 2 | 1 |
| DAY 7 | n | 6 | 6 | 8 | 8 | 8 | 8 | 7 | 7 |
|  | Mean | 0.3 | -0.7 | 0.3 | 0.1 | 1.4 | -0.9 | 0.6 | -0.4 |
|  | SD | 0.82 | 0.52 | 0.46 | 0.35 | 2.00 | 0.99 | 0.79 | 0.53 |
|  | CV% | 244.9 | -77.5 | 185.2 | 282.8 | 145.1 | -113.3 | 137.7 | -124.7 |
|  | Minimum | 0 | -1 | 0 | 0 | 0 | -3 | 0 | -1 |
|  | Median | 0.0 | -1.0 | 0.0 | 0.0 | 1.0 | -1.0 | 0.0 | 0.0 |
|  | Maximum | 2 | 0 | 1 | 1 | 6 | 0 | 2 | 0 |
| DAY 8 | n | 6 | 6 | 8 | 8 | 8 | 8 | 7 | 7 |
|  | Mean | 0.7 | -0.3 | 0.1 | 0.0 | 1.6 | -0.6 | 0.4 | -0.6 |
|  | SD | 0.82 | 0.52 | 0.35 | 0.00 | 2.07 | 0.74 | 0.53 | 0.53 |
|  | CV% | 122.5 | -154.9 | 282.8 | 0 | 127.1 | -119.0 | 124.7 | -93.5 |
|  | Minimum | 0 | -1 | 0 | 0 | 0 | -2 | 0 | -1 |
|  | Median | 0.5 | 0.0 | 0.0 | 0.0 | 1.0 | -0.5 | 0.0 | -1.0 |
|  | Maximum | 2 | 0 | 1 | 0 | 6 | 0 | 1 | 0 |
| DAY 9 | n | 6 | 6 | 8 | 8 | 8 | 8 | 7 | 7 |
|  | Mean | 0.5 | -0.5 | 0.1 | 0.0 | 1.9 | -0.4 | 0.4 | -0.6 |
|  | SD | 0.84 | 0.55 | 0.35 | 0.00 | 2.59 | 0.92 | 0.53 | 0.53 |
|  | CV% | 167.3 | -109.5 | 282.8 | 0 | 138.0 | -244.3 | 124.7 | -93.5 |
|  | Minimum | 0 | -1 | 0 | 0 | 0 | -2 | 0 | -1 |
|  | Median | 0.0 | -0.5 | 0.0 | 0.0 | 1.0 | 0.0 | 0.0 | -1.0 |
|  | Maximum | 2 | 0 | 1 | 0 | 8 | 1 | 1 | 0 |
| DAY 10 | n | 6 | 6 | 8 | 8 | 8 | 8 | 7 | 7 |
|  | Mean | 0.7 | -0.3 | 0.4 | 0.3 | 2.0 | -0.3 | 0.4 | -0.6 |
|  | SD | 1.21 | 0.82 | 0.52 | 0.46 | 2.62 | 0.71 | 0.53 | 0.53 |
|  | CV% | 181.7 | -244.9 | 138.0 | 185.2 | 130.9 | -282.8 | 124.7 | -93.5 |
|  | Minimum | 0 | -1 | 0 | 0 | 0 | -1 | 0 | -1 |
|  | Median | 0.0 | -0.5 | 0.0 | 0.0 | 1.0 | 0.0 | 0.0 | -1.0 |
|  | Maximum | 3 | 1 | 1 | 1 | 8 | 1 | 1 | 0 |
| DAY 11 | n | 6 | 6 | 8 | 8 | 8 | 8 | 7 | 7 |
|  | Mean | 0.8 | -0.2 | 0.9 | 0.8 | 2.0 | -0.3 | 0.7 | -0.3 |
|  | SD | 1.17 | 0.75 | 1.36 | 1.39 | 2.62 | 0.71 | 0.76 | 0.95 |
|  | CV% | 140.3 | -451.7 | 155.0 | 185.2 | 130.9 | -282.8 | 105.8 | -332.9 |
|  | Minimum | 0 | -1 | 0 | 0 | 0 | -1 | 0 | -1 |
|  | Median | 0.5 | 0.0 | 0.5 | 0.0 | 1.0 | 0.0 | 1.0 | -1.0 |
|  | Maximum | 3 | 1 | 4 | 4 | 8 | 1 | 2 | 1 |
